# Reduced B12 uptake and increased gastrointestinal formate are associated with archaeome-mediated breath methane emission in humans

**DOI:** 10.1186/s40168-021-01130-w

**Published:** 2021-09-24

**Authors:** Christina Kumpitsch, Florian Ph. S. Fischmeister, Alexander Mahnert, Sonja Lackner, Marilena Wilding, Corina Sturm, Anna Springer, Tobias Madl, Sandra Holasek, Christoph Högenauer, Ivan A. Berg, Veronika Schoepf, Christine Moissl-Eichinger

**Affiliations:** 1 Diagnostic and Research Institute of Hygiene, Microbiology and Environmental Medicine, Medical University of Graz, Neue Stiftingtalstraße 6, 8010 Graz, Austria; 2 Department of Psychology, University of Graz, 8010 Graz, Austria; 3 Department of Biomedical Imaging and Image-guided Therapy, Medical University of Vienna, 1090 Vienna, Austria; 4 Division of Immunology and Pathophysiology, Medical University of Graz, 8010 Graz, Austria; 5 Gottfried Schatz Research Center for Cell Signaling, Metabolism and Aging, Molecular Biology & Biochemistry, Medical University of Graz, 8010 Graz, Austria; 6 BioTechMed, 8010 Graz, Austria; 7 Division of Gastroenterology and Hepatology, Medical University of Graz, Graz, Austria; 8 Institute for Molecular Microbiology and Biotechnology, University of Münster, Münster, Germany

**Keywords:** Archaeome, Microbiome, Methanogens, Methane, Gut, Gastrointestinal tract, Metabolome, Metagenome, *Methanobrevibacter*, Christensenellaceae

## Abstract

**Background:**

Methane is an end product of microbial fermentation in the human gastrointestinal tract. This gas is solely produced by an archaeal subpopulation of the human microbiome. Increased methane production has been associated with abdominal pain, bloating, constipation, IBD, CRC or other conditions. Twenty percent of the (healthy) Western populations innately exhale substantially higher amounts (>5 ppm) of this gas. The underlying principle for differential methane emission and its effect on human health is not sufficiently understood.

**Results:**

We assessed the breath methane content, the gastrointestinal microbiome, its function and metabolome, and dietary intake of one-hundred healthy young adults (female: *n* = 52, male: *n* = 48; mean age =24.1). On the basis of the amount of methane emitted, participants were grouped into high methane emitters (CH_4_ breath content 5–75 ppm) and low emitters (CH_4_ < 5 ppm).

The microbiomes of high methane emitters were characterized by a 1000-fold increase in *Methanobrevibacter smithii*. This archaeon co-occurred with a bacterial community specialized on dietary fibre degradation, which included members of Ruminococcaceae and Christensenellaceae. As confirmed by metagenomics and metabolomics, the biology of high methane producers was further characterized by increased formate and acetate levels in the gut. These metabolites were strongly correlated with dietary habits, such as vitamin, fat and fibre intake, and microbiome function, altogether driving archaeal methanogenesis.

**Conclusions:**

This study enlightens the complex, multi-level interplay of host diet, genetics and microbiome composition/function leading to two fundamentally different gastrointestinal phenotypes and identifies novel points of therapeutic action in methane-associated disorders.

Video Abstract

**Supplementary Information:**

The online version contains supplementary material available at 10.1186/s40168-021-01130-w.

## Background

Methane is the metabolic end-product of a non-bacterial sub-population of the gastrointestinal microbiome, namely the archaeome [1]. Although methane is not utilized by the human itself, elevated methane levels, measured in breath, have been linked with small intestinal bacterial overgrowth, colorectal cancer, diverticulosis and other gastrointestinal disorders (summarized in [2]). While its role as a gasotransmitter is controversially discussed [3], methane is causally linked to a slowed gastrointestinal motility (transit time slowed down by up to 59%), probably caused by the direct action of methane on the cholinergic pathway of the enteric nervous system [4].

Methane-forming archaea (‘methanogens’) in the gastrointestinal tract (GIT) were first observed long ago—through the detection of methane in the human breath and flatus (see also [5, 6]). Although not a single pathogenic archaeal representative has been identified, human-associated archaea are widespread in the GIT as well as other body sites (e.g. skin, respiratory tract) [1, 7, 8]. The role of methanogens per se in health and disease is not yet clear, and analyses suffer from methodological pitfalls to correctly detect and characterize the human archaeome as well as the contradictory information that appears in the literature (reviewed in [1]).

Although the average abundance of archaea in human fecal samples is low as compared to bacteria [1], methanogens are considered to represent key-stone species in the GIT. By maintaining numerous syntrophic relationships with bacteria, methanogens control the efficiency of the bacterial primary and secondary fermentation of complex organic molecules. By consuming by-products of bacterial metabolism (H_2_, CO_2_, formate, methyl-compounds, acetate), they particularly contribute to keeping the hydrogen concentration low, which would inhibit the fermentation activity and reduce the overall energy yield [1].

In the human GIT, methanogens are mainly represented by the Methanobacteriales (*M. smithii, Methanosphaera stadtmanae*) and Methanomassiliicoccales (*Ca.* Methanomassiliicoccus and *Ca.* Methanomethylophilus representatives). These methanogens contribute to an average human body methane emission of about 0.35 l per day [9], released through the breath and flatus. In general, clinical breath tests (mainly focussing on hydrogen content) are widely distributed in clinical diagnosis of gastrointestinal conditions, including irritable bowel syndrome, maldigestion or small intestinal bacterial overgrowth [10]. Based on such breath tests, increased methane content has been associated in some reports with colorectal cancer or diverticulosis [2]. However, a substantial proportion of the human population (approx. 20% of the Western adult population) has been shown to naturally emit methane in concentrations above 5 ppm, measured in breath, whereas the remaining population emits methane in concentrations close to or below the detection limit (for details see [1]). Although increased methane emission has been linked to the successful cultivation and increased molecular detection of methanogenic archaea from stool [11, 12], the underlying reason for archaeal differential abundance in human methane producers and non-producers is largely unclear to date.

In this publication, we identify the driving forces supporting methane emission through breath by a systematic comparison of high methane-emitting young subjects vs. low-emitters with respect to diet, GIT microbiome and archaeome (amplicon- and shotgun metagenome-based analyses), and metabolome.

## Methods

All key resources and PCR conditions are listed in the Supplementary Methods file.

### Subject details

One-hundred participants between 18 and 37 years were recruited at the University of Graz. Following exclusion criteria were set: smoker, intake of antibiotics and probiotics within the last 3 months before sampling and neurological, psychiatric or internal diseases. The study was evaluated and approved according to the Declaration of Helsinki by the local ethics committee of the University of Graz (EK-Nr. GZ. 39/44/63 ex 2017/18). Before participation, all participants signed an informed consent.

### Methane measurement

All volunteers were asked to inhale deeply through the nose and hold their breath for 15 s before complete exhalation into the GastroCH_4_ECK breath bags (Bedfont Scientific Ltd., UK). Breath was collected on the same day as the stool sample in the morning before brushing their teeth and eating breakfast. Methane in the breath was measured by GastroCH_4_ECK Gastrolyzer (Bedfont Scientific Ltd., UK). In order to define a cut-off value for high- and low-methane-producing individuals, we adopted a conservative cut-off, proposed after analysis of a large North American dataset of methane measurements in breath (4–5 ppm [13]). Based on these considerations, participants with CH_4_ values above 5 ppm were stated as methane producers in our study. The median of high-methane-producing individuals was 14 ppm (ranging from 7 to 75 ppm), whereas the median of the low-methane-emitting individuals was found to be 1 ppm (ranging from 1 to 4 ppm; Supplementary Table 1). With these measurements, 15% of the study group (*n*=15) were classified as high methane emitters (CH_4_ value ≥ 5 ppm).

### Matched subset (*n*=30)

Fifteen high-methane emitters were matched to 15 low-methane emitters by sex (same sex), age (max. 7 years difference), hormonal contraception (both either yes or no), and vegetarianism (both either yes or no) (Supplementary Table 2, column D-H). All other participants were excluded in this subset.

### Nutritional assessment

Dietary habits and food intake information of the 4 weeks before the investigation were collected by a validated food frequency questionnaire (‘German Food Frequency Questionnaire (FFG)’ of the Robert Koch Institute) [14]. The diet’s nutritive composition (e.g. intake of fat, protein, magnesium, zinc, etc.) and dietary diversity indices were analyzed by a specific nutrition software using food and nutritive values specific for Austria [15]. The dietary intake information is included in Supplementary Table 2.

### Sample collection, DNA extraction and amplicon sequencing

#### Collection and PMA treatment

Every participant had to collect a stool sample in a stool collection tube (VWR) and bring it to the laboratory. After arrival, stool samples were placed on ice immediately. Before storage at −20°C, samples were preprocessed with propidium monoazide (PMA) to make sure that we analyze intact cells. Therefore, a 10% stool (0.1g stool) suspension with 0.9% sodium chloride was treated with PMA solution to mask freely accessible DNA. During PMA treatment, all steps were performed in the dark. PMA solution (final concentration: 50 μM) was added to the stool samples. Samples were vortexed briefly, incubated for 10 min on a shaker and 15 min in a PMA-Lite™ LED Photolysis Device (Biotum) afterwards. Samples were stored at −20°C until further use.

#### DNA extraction

300 μl of PMA-treated stool samples were used to extract microbial genomic DNA by using the DNeasy PowerSoil Kit (QIAGEN, USA) according to manufacturer’s protocol. The only modification was the use of MagNaLyser at 6500 rpm for 2 times 30 s instead of vortexing the samples. DNA concentration of extracted DNA was quantified via Qubit dsDNA HS Assay Kit (Thermo Fisher Scientific, USA).

#### Quantitative PCR

The absolute number of bacterial and methanogenic 16S rRNA gene copies in the samples was assessed using a SYBR-based procedure. One-microliter template was added to SYBR Green Supermix (BioRad). The primer pairs 331F and 797R and M1F and M1R were used for bacterial and methanogenic (mcrA gene) qPCR, respectively. The PCR reagents and conditions are given in the Supplementary Methods file.

Crossing point (Cq) values were determined by the Bio-Rad CFX Manager Software version 3.1 (regression method). Absolute copy numbers of bacterial and methanogenic 16S rRNA genes were calculated using the Cq values and the reaction efficiencies based on standard curves obtained from defined DNA samples from *Escherichia coli* and the gene of the alpha subunit of the methyl coenzyme M reductase (mcrA) [16, 17]. The average Cq values of our non-template controls were used to define the detection limits. All reactions have been performed in triplicates. For further analysis, only samples with positive results in at least 2 out of 3 replicates were used. The qPCR efficiency and *R*^2^ values yielded 93.6% and 0.997 in bacterial approach and 70.0% and 0.985 in methanogen-targeting approach, respectively.

#### 16S rRNA gene-based next-generation sequencing (NGS) and sequence data processing

To determine the microbial diversity, the variable region V4 of 16S rRNA gene was amplified using universal PCR primers 515FB and 806RB. For the archaea-targeted set-up, a nested PCR approach was used, using the primer pair 344F and 1041R at the first and 519F and 806R for the second PCR. For detailed protocol and primer sequences, see [18]. Each PCR reaction was performed in triplicates. Triplicates were pooled after visualization in 3% (w/v) agarose gel. Fragments were sequenced using the Illumina MiSeq sequencing platform (Illumina, Eindhoven, the Netherlands) performed in cooperation with the Core Facility for Molecular Biology of the Center for Medical Research in Graz [19].

Raw reads were analyzed with QIIME2 (Quantitative Insights Into Microbial Ecology) version 2019.1 using DADA2 (Divisive Amplicon Denoising Algorithm) to denoise sequences [20, 21]. Briefly, paired end reads were joined together before a quality check of the produced sequences was performed. Afterwards, taxonomic assignment was realized with a Naïve-Bayes classifier trained on the SILVA v128 (universal approach) and SILVA v132 (archaeal approach) reference database [22, 23]. For phylogenetic metrics and analysis, a rooted tree was generated with FastTree 2 [24].

LEfSe (LDA Effect Size) [25] was used to identify features characterizing the differences between two given conditions. In our case, the LEfSe tool was integrated in a user-friendly Galaxy set-up provided by the Core Facility Computational Biology at the Medical University of Graz. The cladogram was created by the ‘Plot Cladogram’ function and curated using Inkscape (inkscape.org).

#### Controls

Extraction blanks and PCR negative controls were processed in parallel. All controls were removed using the R package decontam [26] with the prevalence method and threshold set to 0.5 (https://github.com/benjjneb/decontam). Unassigned sequences mitochondrial and chloroplast signatures as well as features with zero or only one read were also removed. Remaining RSV tables (Supplementary Datasets 1, 2 and 3) were processed in Calypso [27] to generate RDA, Shannon, PCoA, ANOVA plots as well as co-occurrence plots based on Spearman correlation analysis.

### BioEnv

R Studio version 1.2.1335 (2018-07-02) and R package vegan 2.5-5 [28] was used to generate a BioEnv diagram with environmental variables (dietary information, CH_4_ emission) with a maximum correlation with microbial community dissimilarities.

### Metagenome analysis

#### Shotgun metagenome sequencing

200 ng extracted DNA (PMA treated) of each of the 30 matched samples was sent for sequencing to Macrogen (Seoul, South Korea). Library was extracted via Nextera XT Library construction kit (Illumina, Eindhoven, the Netherlands; Library Reference Guide: #15031942 v03) and sequenced without a prior ribosomal depletion step (150 bp paired end) using one lane with the Illumina HiSeq platform (Illumina, Eindhoven, the Netherlands). Fastq files were received as output after sequencing. On average, 2,741,962 ± 795,487 sequences per sample were obtained (Supplementary Table 8).

#### Metagenomics analysis via MG-Rast

Raw data (fastq files) was quality controlled, and sequences were paired and analyzed with the open-submission data MG-Rast platform (server running version 4.0.3.) [29]. 85.99 ± 4.1% (1,890,579 ± 536,553 sequences) of the obtained reads were successfully mapped (Supplementary Table 8). Features with zero or one read were removed before feature tables (RefSeq and SEED) were uploaded in Calypso [27].

#### Metagenome assembled genomes (MAGs)

After checking quality with fastqc (v0.11.8) [24], raw shotgun reads were filtered accordingly with trimmomatic (v0.38) [30] by using a minimal length of 50 bp and a Phred quality score of 20 in a sliding window of 5 bp. Quality-filtered sequences were then mapped against the human chromosome hg19 with bowtie2 (v2.3.5) [31] to remove sequences of the human host by retaining all unmapped reads with samtools (v1.9, settings: -b -f 12 -F 256) [32]. Host removed forward and reverse fastq files were then extracted from sorted bam files with bedtools (v2.29.0) [33]. Reads were then analyzed in a genome-centric manner. In a first step, quality-filtered reads were co-assembled in Megahit (v1.1.3) [34] by using the preset meta-sensitive. Resulting contigs were binned with MaxBin v2.2.4 [35]. Further on, bins were quality scored (based on CheckM [36] estimates for completeness, contamination and strain heterogeneity as well as N50 based assembly continuity) and de-replicated to pick representative MAGs (metagenome assembled genomes) with dRep (v2.0.5) [37]. Quality MAGs were then classified with GTDBtk (v1.2.0) [38]. Identified key MAGs were further annotated and analyzed including gene synteny in MaGe [39]. Finally, replication rates were determined with iRep (v1.1.9) [40].

### Prediction model, supervised metadata classifications and regressions

Raw metagenome data was used to create prediction models in QIIME2 [41]. The q2-sample-classifier-plugin [42] was used to predict high- and low-methane emitters from feature table compositions. To determine accuracy by comparing predicted values, the data set was randomly split by 5 into a training set (4/5) and a test set (1/5). The training set was used for the learning model including settings for optimized feature-selection, parameter tuning and K-fold cross-validation based on RandomForest. The resulting sample estimator (trained classification model) was also used to predict methane emissions between the shotgun (RefSeqs) and amplicon dataset.

### Krona charts

Datasets (amplicon and metagenome) were normalized and Krona chart templates [43] were used to visualize the differences between high- and low-methane emitters.

### Metabolic quantification using NMR

Nuclear magnetic resonance spectroscopy (NMR) analysis was used to analyze concentrations of acetate, succinate, formate, lactate, butyrate and propionate in stool samples (PMA untreated) performed at the Gottfried Schatz Research Center for Cell Signaling, Metabolism and Aging, Molecular Biology and Biochemistry, Medical University of Graz. To quench enzymatic reactions and remove proteins, methanol-water solution was added to the stool sample (2:1), cells were lysed using a Precellys homogenizer and stored at −20°C for 1 h until further processing. Samples were centrifuged (4°C, 30 min, 17949 rcf), and supernatants were lyophilized afterwards. Samples were then mixed with 500 μl NMR buffer in D_2_O (0.08 M Na_2_HPO_4_, 5 mM 3-(trimethylsilyl) propionic acid-2,2,3,3-d_4_ sodium salt (TSP), 0.04 (w/v) % NaN_3_ in D_2_O, pH adjusted to 7.4 with 8 M HCl and 5 M NaOH) and transferred into 5-mm NMR tubes. NMR was performed on an AVANCE™ Neo Bruker Ultrashield 600 MHz spectrometer equipped with a TXI probe head at 310 K and processed as described elsewhere [44].

The 1D CPMG (Carr-Purcell_Meiboom_Gill) pulse sequence (cpmgpr1d, 512 scans, 73728 points in F1, 11904.76 HZ spectral width, 512 transients, recycle delays 4 s) with water suppression using pre-saturation was used for ^1^H 1D NMR experiments. Bruker Topspin version 4.0.2 was used for NMR data acquisition. The spectra for all samples were automatically processed (exponential line broadening of 0.3 Hz), phased and referenced using TSP at 0.0 ppm using Bruker Topspin 4.0.2 software (Bruker GmbH, Rheinstetten, Germany).

Spectra pre-processing and data analysis have been carried out using the state-of-the-art data analysis pipeline (group of Prof. Jeremy Nicholson at Imperials College London) using Matlab® scripts and MetaboAnalyst 4.0 [45]. NMR data were imported to Matlab® vR2014a (Mathworks, Natick, Massachusetts, USA), regions around the water, TSP, and remaining methanol signals excluded, and to correct for sample metabolite dilution probabilistic quotient normalization [46] was performed.

Stated concentrations correspond to normalized concentrations after probabilistic quotient normalization. Concentrations of metabolites of interest are found in Supplementary Table 5.

### Metabolic predictions

Potential metabolites were predicted with the q2-micom plugin (v. 0.8.0) [47]. All analysis were conducted with the AGORA genus model database (v1.03) [48] and covered the entire dataset (*n*=100) and the matched dataset (*n*=30) as well as all and selected key features. In addition, the standard western diet gut medium was adapted with the help of these tutorials (https://github.com/micom-dev/media and https://micom-dev.github.io/micom/media.html) according to measured nutrients to provide a per sample diet model as well. No abundance cutoff was used for all and selected features. In addition, a leave one out strategy was included for selected features to determine the behaviour of the established metabolic models in the absence of a potential microbial key-player. The growth simulation was performed with individual settings for the tradeoff between community growth rate and individual taxon growth rate. This pressure to the model was determined by an evaluation of the tradeoff from 0 to 1 (zero to maximum enforced growth) and was set between 0.1 and 0.7 accordingly (all features and selected features, respectively). Resulting growth rates could be partly verified with calculated replication rates using iRep of representative key MAGs. Subsequent visualizations and analysis included potential metabolite consumptions, growth niches and metabolite fluxes in dependence of measured methane emissions for all datasets; however, for improved clarity, the displayed metabolite flux analysis (Fig. 7) was based on 16S rRNA gene amplicons of the matched cohort (*n*=30) and selected keystone taxa. Finally, a minimal medium was determined for selected key features of matched samples.

### Quantification and statistical analysis

Statistical tests (Spearman rho’s and Pearson’s correlation) were performed using IBM SPSS Amos version 26. Different parameters were checked for normal distribution. Correlations were calculated based on distribution of the compared parameters via Spearman’s rho and Pearson’s correlation, respectively. In the manuscript, non-corrected *p* values were used to describe specific trends; however, Bonferroni corrected *p* values can be found in Supplementary Table 6.

### Data and software availability

Raw sequencing data obtained from amplicon-based sequencing and metagenomics sequencing data (technical sequences including adaptor sequences, linker sequences and barcode sequences as well as human reads were removed) used in this paper can be found in the European Nucleotide Archive (ENA): PRJEB41867. Supplementary Datasets (after decontam and removal of features with zero and one reads) and all Supplementary figures, tables and items were deposited on Mendeley at 10.17632/hjj3tx7n84.1. Software and algorithms used are listed in detail in the Supplementary Methods file.

## Results

### Study overview

In total, 100 participants (female: *n* = 52, male: *n* = 48; mean age =24.1) were recruited in this study. Metadata information (sex, age, vegetarian yes/no, contraception yes/no, breath methane content as well as metabolite information) of all participants is provided in Supplementary Table 1. All participants provided one stool sample, one breath sample for methane measurements and a completed dietary questionnaire. Based on the amount of methane emitted, participants were grouped into high-methane emitters (HE; CH_4_ value: 5–75 ppm) and low emitters (LE; CH_4_ value < 5 ppm). Fifteen percent of the participants were categorized as HEs (Supplementary Table 2), with the percentage in congruence with known levels of methane emission of young adult European cohorts [9]. For specific scientific questions, 15 high-methane emitters were matched to 15 low-methane emitters by sex, age, hormonal contraception and vegetarianism (Supplementary Table 2; *n*=30).

The following data sets were obtained: ‘universal’ and archaeal 16S rRNA gene profiles for all stool samples, a metagenomics dataset as well as metabolomic information (e.g. acetate, succinate, formate) and detailed dietary information (e.g. diversity, energy, protein, fat, carbohydrates) from matched participants (Supplementary Datasets 1, 2, 3 and 4; Supplementary Tables 1, 2 and 5).

### High-methane microbiomes are characterized by a specific microbial community and a 1000-fold increase in *Methanobrevibacter* signatures

**Fig. 1:**
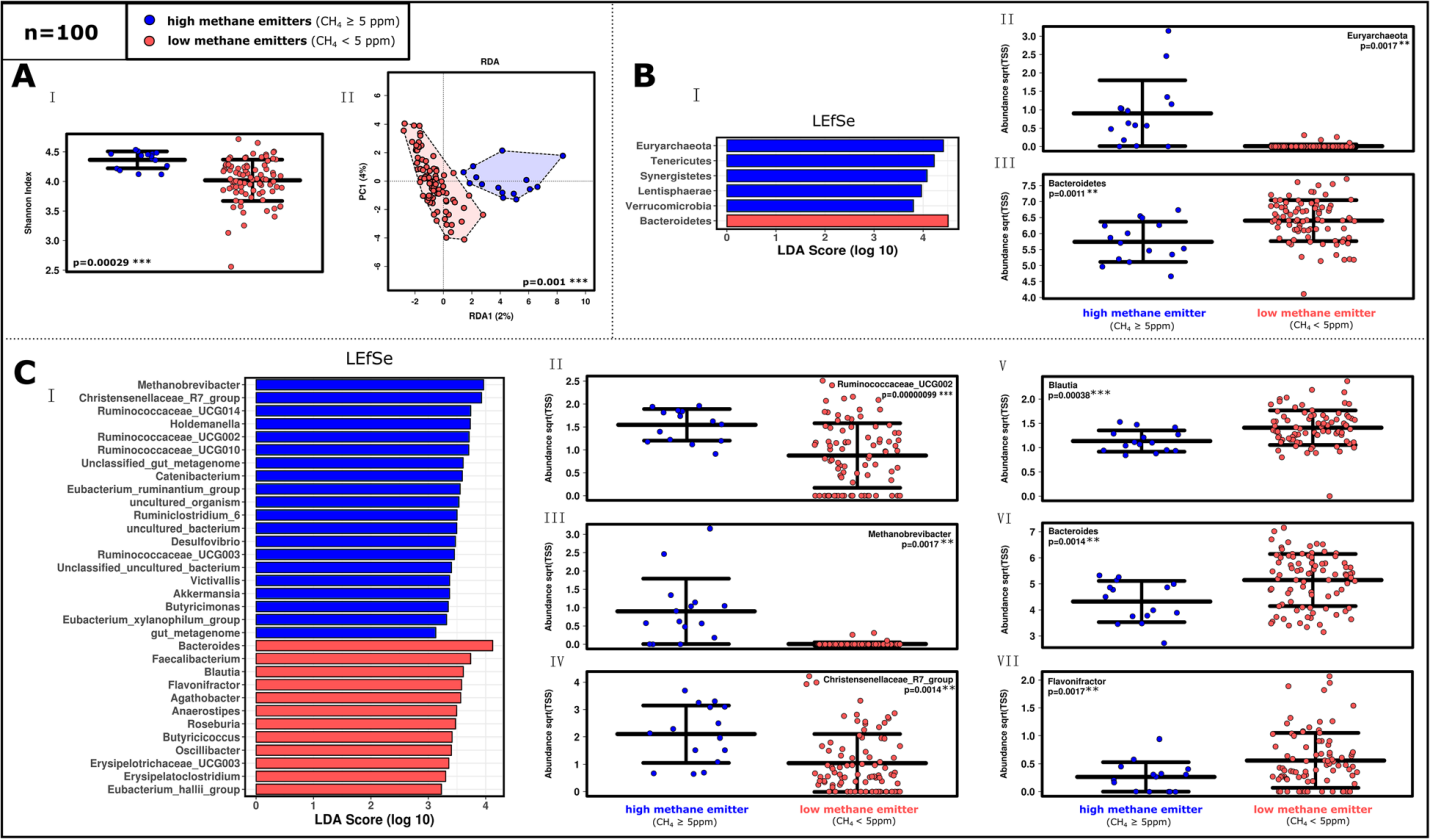
Differences in alpha and beta diversity based on the ‘universal’ approach of 16S rRNA gene sequencing between high (HE) and low-methane emitters (LE). Profiles of the whole study cohort (***n***=100) are shown. The profiles of the matched study subset (***n***=30) are shown in Supplementary Figure 1. **A.I.** An examination of Shannon diversity index revealed significant differences in alpha diversity (RSV (ribosomal sequence variants) based; analysis of variance, ANOVA). **A.II.** The microbiome of HEs clustered significantly differently in the RDA plot (RSV based). **B.I** LEfSe (Linear Discriminant Analysis Effect Size) analysis of the 100 most abundant phyla and **B.II–III.** Relative abundance of selected phyla in ANOVA plots. **C.I.** LEfSe analysis of the 100 most abundant genera. LEfSe determines taxonomic features which are most likely to explain differences between groups by coupling tests for statistical significance with other tests for biological consistency and effect relevance [25]. **C.II–VII.** ANOVA plots of selected genera and statistical significance

**Fig. 2:**
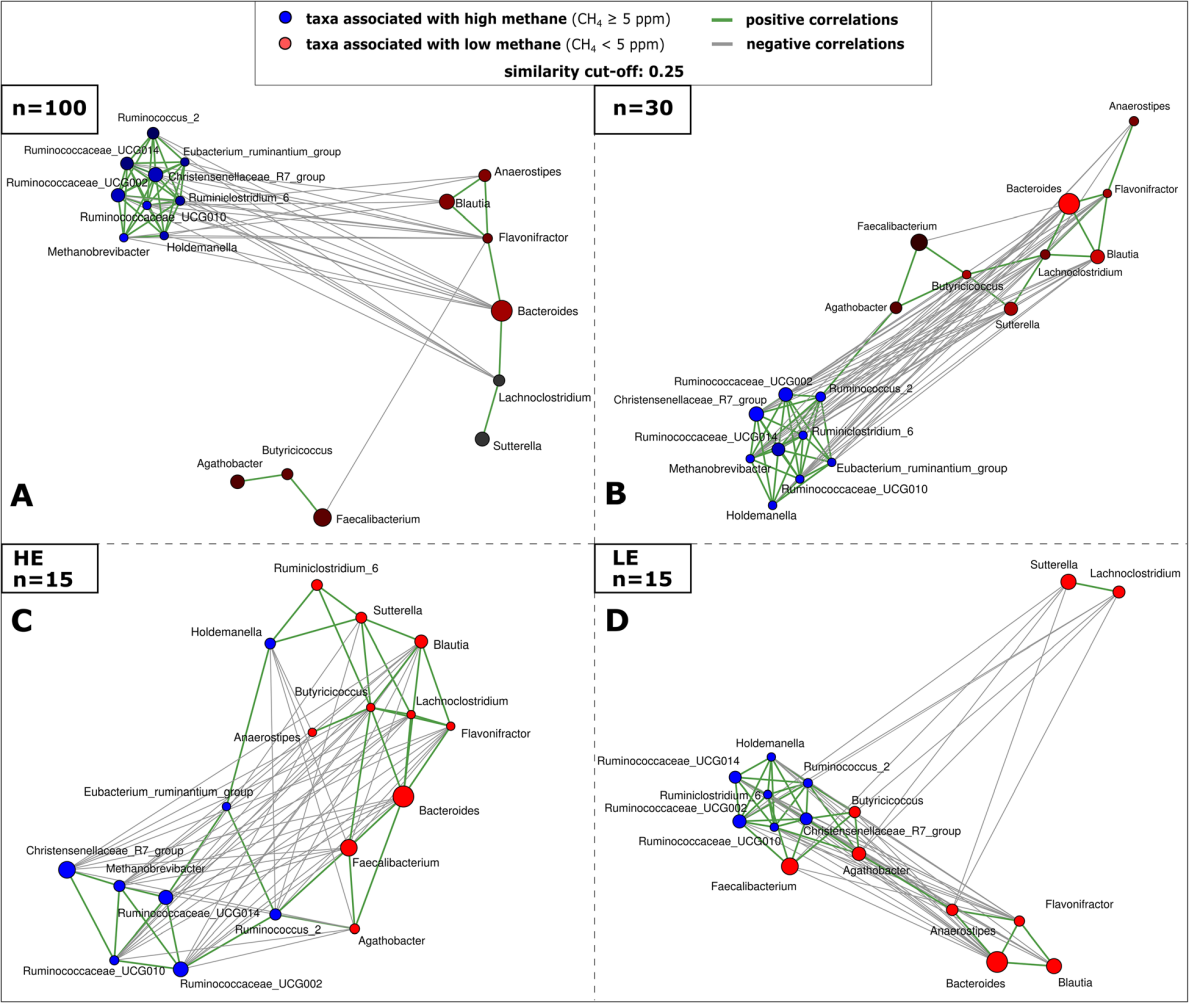
Co-occurrence networks based on Spearman’s rho correlation of selected genera in HE and LE microbiome samples. Taxa were selected based on significantly different relative abundances in both sample types and LEfSe analyses. Left, upper panel: Whole study cohort (*n*=100), right, upper panel: matched study subset (*n*=30). Lower panels show co-occurrence patterns in the HE (left) or the LE samples (right)

The microbiomes of high-methane emitting subjects (HEs) were characterized by significantly higher alpha diversity (Fig. 1A.I) and a substantially different microbiome composition, compared to low-methane-emitting persons (LEs). Although the HE microbial profiles did not group separately in the PCoA plot (Supplementary Figure 1A.I), the parameter ‘methane production’ had a significant impact on the microbiome composition in redundancy analysis (RDA; Fig. 1A.II). Methane-emitting microbiomes were significantly associated with Euryarchaeota (*Methanobrevibacter*) and signatures of Christensenellaceae R7 group, which formed a stable network with different *Ruminococcus*/Ruminococcaceae, *Holdemanella* and the *Eubacterium ruminantium* group (Fig. 2). On the contrary, LEs were characterized by a predominance of Bacteroidetes, and a stable network of *Bacteroides, Lachnoclostridium, Sutterella, Flavonifractor, Blautia* and *Anaerostipes* (Figure 1B-C,2; Supplementary Figures 1, 2 and 3). A Krona Chart overview of the taxonomic composition of HE and LE samples is provided in Supplementary Item 1, displaying the 1000-fold increase of relative abundance of *Methanobrevibacter* signatures in high methane emitters (HE: 2%, LE: 0.002%; for comparison: *Bacteroides* (HE: 19%, LE: 28%), Christensenellaceae R7 group (HE: 6%, LE 2%), and *Ruminococcaceae UCGs* (HE: 22%, LE: 20%)). Notably, a similar increase of archaeal signatures compared to overall bacterial 16S rRNA gene copies was observed using quantitative PCR (HE: 0.78%, LE: 0.002%; Supplementary Figure 4; Supplementary Table 7).

**Fig. 3:**
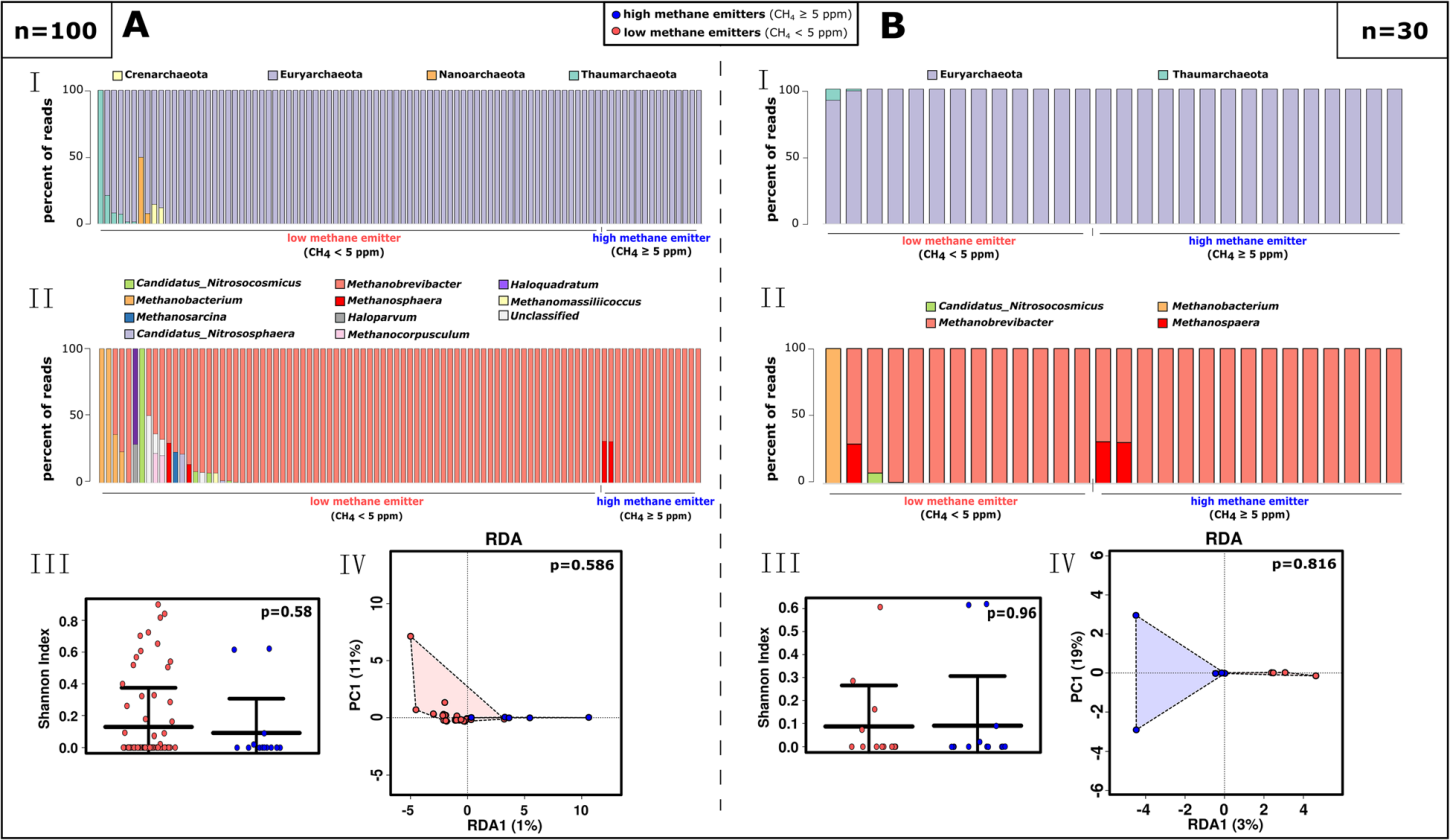
Archaeome profile of HE and LE samples, based on the ‘archaeal’ approach of 16S rRNA gene sequencing. **A** Profile of the whole study cohort (*n*=100). **B** Matched study subset (*n*=30). **I.** Bar chart of the 20 most abundant taxa compared regarding their low- or high-methane emission at the phylum level and **II.** at the genus level. **III.** Shannon diversity and **IV.** RDA plot at RSV level

Notably, methane emission and the associated increase of *Methanobrevibacter* signatures were solely driven by a single *M. smithii* ribosomal sequence variant (RSV; Supplementary Dataset 2). Besides that, the archaeal communities of high- and low-methane emitters were not significantly different with respect to their alpha or beta diversity (Fig. 3). Samples from high methane emitters did not contain any archaeal signatures apart from the Euryarchaeota, i.e. *Methanobrevibacter* and *Methanosphaera*. In the entire dataset, 21 *Methanobrevibacter* RSVs were observed, whereas *Methanosphaera* was represented by only two RSVs (both genera are represented by one RSV each in the universal dataset).

The microbiome profile of the matched study subset (*n*=30) was highly similar to the profiles revealed for the non-matched volunteers, and the same characteristics, with respect to microbiome composition, alpha diversity, co-occurrences, etc., was observed (Supplementary Dataset 1; Supplementary Figure 1B, 2B, 3B-D, 5; Fig. 2; Fig. 3B)

### High-methane emitter microbiomes are specialized on C1–C3 compound turnover

The functional analysis of the metagenomics dataset was based on 14,616,890 sequences, which were categorized into 28 SEED subsystems and contained 6956 actual function assignments and 6589 unique features. The output was organised hierarchically into four levels; level one represented the SEED subsystem and level four represented the most detailed functional information.

**Fig. 4:**
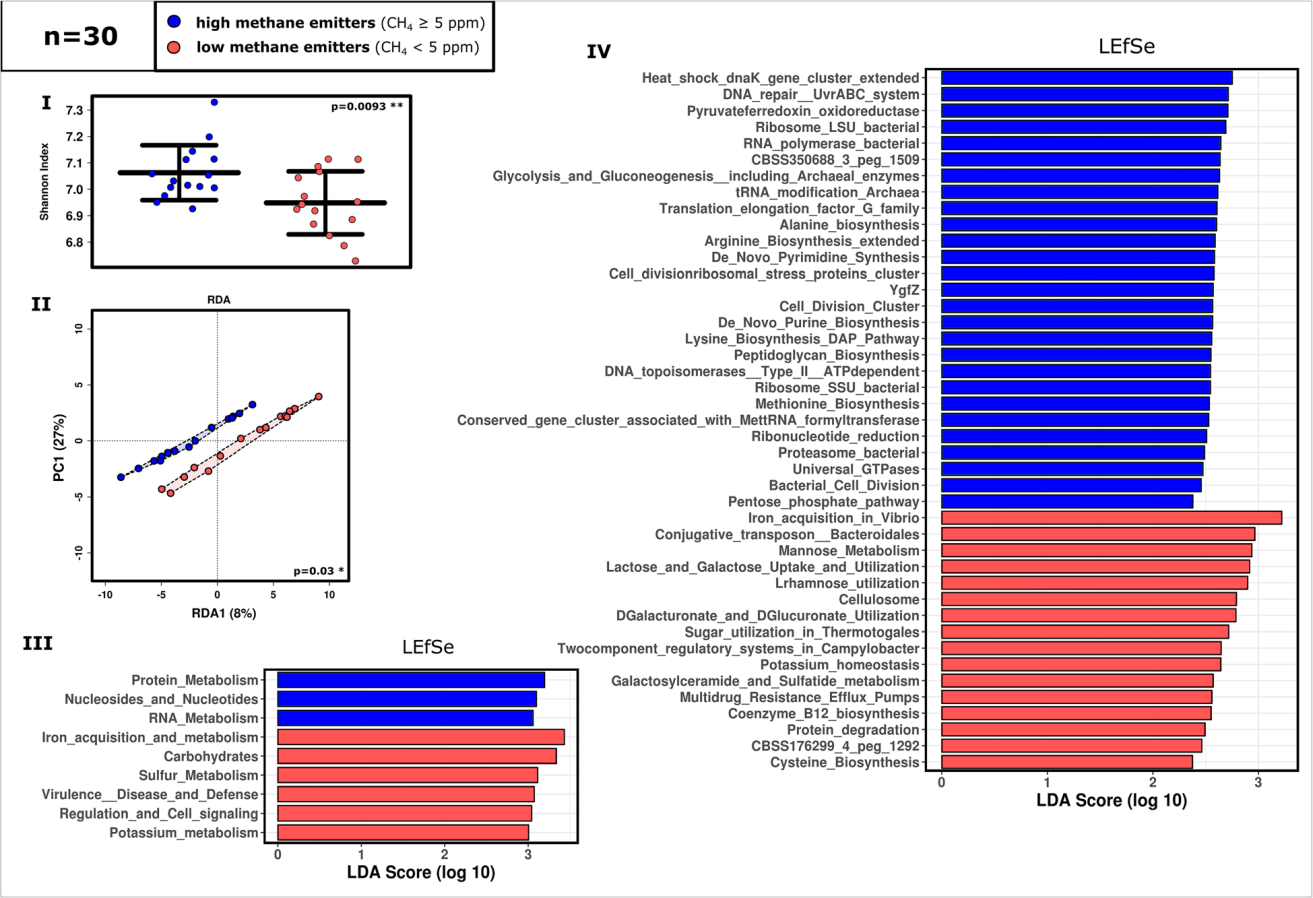
Overview of the divergent functions of the HE and LE based on the shotgun metagenome analysis (subsystems). **I.** Shannon diversity and **II.** RDA plot at feature level. **III. and IV.** LEfSe analysis at level 1 (highest subsystem level) and level 3, respectively. (100 most abundant; *n*=30); ANOVA plots of selected functions are given in Supplementary Figure 7

An overview on the detected functions is available in Supplementary Item 3 and Supplementary Figure 6 (Supplementary Dataset 3). Like the profile information derived from 16S rRNA gene data, the diversity of unique functions was significantly higher in samples from high-methane emitters as compared to low methane emitters (Fig. 4A). The impact of methane emission on the overall functions was also found to be significant (Fig. 4A). At level 1, LEfSe analysis identified LE microbiomes to be significantly associated with ‘iron acquisition and metabolism’ (*p*=0.007), ‘carbohydrates’ (*p*=0.034) and ‘sulfur metabolism’ (*p*=0.028; all tests: *t* tests due to normal distribution; Fig. 4B; Supplementary Figure 7). Overall, the microbiomes from low-methane emitters were functionally specialized on turnover of C_6_ and C_5_ carbohydrate components. Among the functions associated with ‘carbohydrate,’ a particular increase in the LE dataset was observed in the ‘monosaccharide’ (level 2) turnover-associated genes (*p*=0.009, Mann-Whitney *U*; HE: 3%, LE: 4%) (e.g. in d-galacturonate, l-rhamnose, xylose, l-arabinose and l-fucose metabolism) as well as in the uptake of lactose and galactose (*p*=0.009, *t* test). Especially mannose metabolism (level 3; HE: 0.8%, LE: 1%; *p*=0.026, Mann-Whitney *U*), including the metabolism of alpha-1,2-mannosidase (level 4; HE: 0.6%, LE: 0.9%; *p*=0.015, Mann-Whitney *U*), was found to be increased in LE samples (Supplementary Figure 7). Indeed, gut-associated *Bacteroides* species carry a specific genetic machinery to degrade plant-derived mannans or human high-mannose-type N-glycans, stemming from mucosal secretions and secreted epithelial cells [49, 50].

The microbiomes from high methane emitters, however, were more directed towards the turnover of C_3_- C_1_ compounds. For instance, the ‘pyruvate ferredoxin oxidoreductase’ (HE: 0.4%, LE: 0.3%; alpha and beta subunits; HE: 0.04% LE: 0.01% (*p*=0.026, *t* test) and HE: 0.02% LE: 0.01%, respectively), which is part of the ‘central carbohydrate metabolism’ of pyruvate, propanoate, and butanoate, and the reductive tricarboxylic acid cycle, was found to be increased in HE samples. This enzyme (also known as pyruvate synthase) catalyzes the interconversion of pyruvate and acetyl-CoA and thus is responsible for the incorporation or release of CO_2_ with the help of ferredoxin. Moreover, the functional gene involved in formate efflux transportation were as well increased in high-methane emitter microbiomes (0.02% vs. 0.005%; *p*=0.03, Mann-Whitney *U*) (Supplementary Dataset 3).

Genes involved in ‘methanogenesis’ were almost absent in the LE dataset (0.00004%), but reached a 0.1% overall relative abundance in the HE dataset (*p*=0.0086, Mann-Whitney *U*). This was also reflected by the methyl-coenzyme M reductase, which is responsible for the release of methane in the last step of methanogenesis, and whose alpha subunit was represented in a proportion of 0.01% in the HE dataset but only of 0.00001% in the LE dataset (*p*=0.000012, Mann-Whitney *U*). Notably, genes involved in ‘methanogenesis from methylated compounds’ comprised 0.01% in the HE dataset, and 0.005% in the LE dataset, indicating that a similar proportion of these genes existed in both datasets, largely independent of methane emission (Supplementary Dataset 3).

Taxonomic information derived from shotgun metagenomics was highly similar to the information that was derived from 16S rRNA gene amplicon sequencing and confirmed the differences between high- and low-methane emitter microbiomes (for details see: Supplementary Dataset 4; Supplementary Item 4; Supplementary Figures 8, 9, 10 and 11). Notably, signatures of Christensenellaceae, which were associated with *Methanobrevibacter* occurrence in the amplicon dataset, could not be retrieved from the metagenomics dataset, a phenomenon that has been reported earlier [51]. Network analyses of the archaeome profile in high- and low-methane emitters on the species level revealed again the predominance of *Methanobrevibacter* species under HE conditions (amongst all archaeal signatures 70% *M. smithii*, 1% M. *stadtmanae*), whereas LE samples were characterized by a more diverse but rarely abundant archaeome (9% *M. smithii*, 3% *M. stadtmanae*; Supplementary Figure 12 and 13; Supplementary Dataset 4; Supplementary Item 5).

Of note, using these initial datasets, methane emission above 5 ppm appeared to be predictable from the RefSeq shotgun dataset (up to 100% prediction accuracy) using a sample classification approach. Specifically, we applied supervised learning methods that had been trained on the amplicon and metagenomic datasets. Although the individual datasets were rather small, which increases the risk of overfitting the learning model, the overall prediction accuracies reached 63.6% in case of 16S rRNA gene amplicons and up to 100% for RefSeq in the shotgun dataset. When we applied the latter classification model to the larger dataset from 16S rRNA gene amplicons, the estimators achieved 85% prediction accuracy. Hence, despite the obvious limitations of our classification model due to sample size and likely overfitting, these results indicate that it has a high potential for predicting methane emissions above 5 ppm.

### High-methane emitter keystone taxa drive nutrient break-down towards C1–C3 compounds

**Fig. 5:**
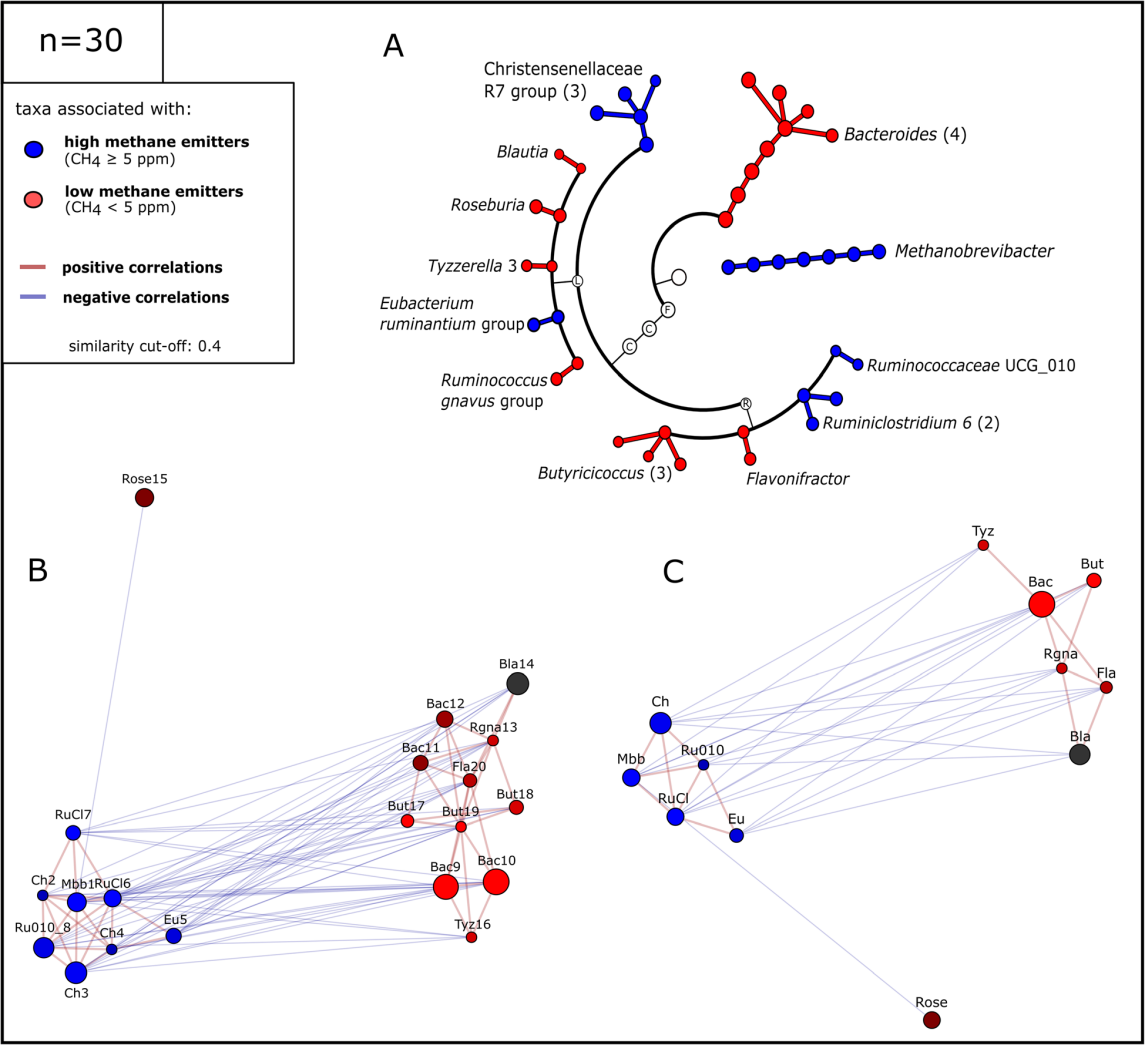
Identified keystone taxa in HE and LE subjects. **A** Cladogram of LE and HE keystone taxa. F Firmicutes, C Clostridia/Clostridiales, L Lachnospiraceae, and R Ruminococcaceae. Numbers in brackets indicate the number of contributing RSVs; **B** and **C** Network of keystone taxa of HE and LE at RSV and genus levels, respectively. Mbb/Mbb1 *Methanobrevibacter,* Ch/Ch2-4 Christensenellaceae R7 group, Eu/Eu5 Eubacterium ruminantium group, Rucl/Rucl6-7 *Ruminiclostridium,* Ru010/Ru010_8 Ruminococcaceaea UCG010, Bac/Bac9-12 *Bacteroides*, Rgna/Rgna13 *Ruminococcus gnavus group*, Bla/Bla14 *Blautia*, Rose/Rose15 *Roseburia*, Tyz/Tyz16 ‘*Tyzzerella*’, But/But17-19 *Butyricicoccus*, Fla/Fla20 *Flavonifractor* (also see Supplementary Table 3)

As indicated above, we identified a number of representative bacterial and archaeal genera, which were indicative for high- and low-methane emission, respectively. To perform more detailed analyses on the RSV level, we proceeded with amplicon data (matched dataset) because taxonomic information for Christensenellaceae was missing from the metagenomics dataset. We identified 21 RSVs, revealing significantly discriminative (identified through LEfSe analyses) and substantial mean abundances (top 600 taxa). We found that the LE profile was mainly defined by four RSVs of *Bacteroides*, four RSVs of *Butyricicoccus* and one RSV each of *Flavonifractor, Blautia*, ‘*Tyzzerella’, Ruminococcus* (*R. gnavus* group), and *Roseburia,* whereas the HE profile was driven by one RSV of *Methanobrevibacter,* three RSVs of the Christensenellaceae R7 group, two RSVs of *Ruminiclostridium,* one RSV of Ruminococcaceae UCG010 and one RSV of *Eubacterium* (*E. ruminantium* group) (Fig. 5, Supplementary Table 3). These taxa are lateron referred to as keystone taxa.

This selection for keystone taxa was further supported by 84 dereplicated high-quality MAGs (metagenome assembled genomes; mean completeness 90%, mean contamination 7%, Supplementary Table 4) with replication rates in the range of 1.3 to 2.6 (*Methanobrevibacter smithii*: 4 MAGs, *Bacteroides:* 32, Christensenellales: 19, Ruminococcaceae: 19, *Ruminiclostridium:* 2, *Ruminococcus:* 4).

**Fig. 6:**
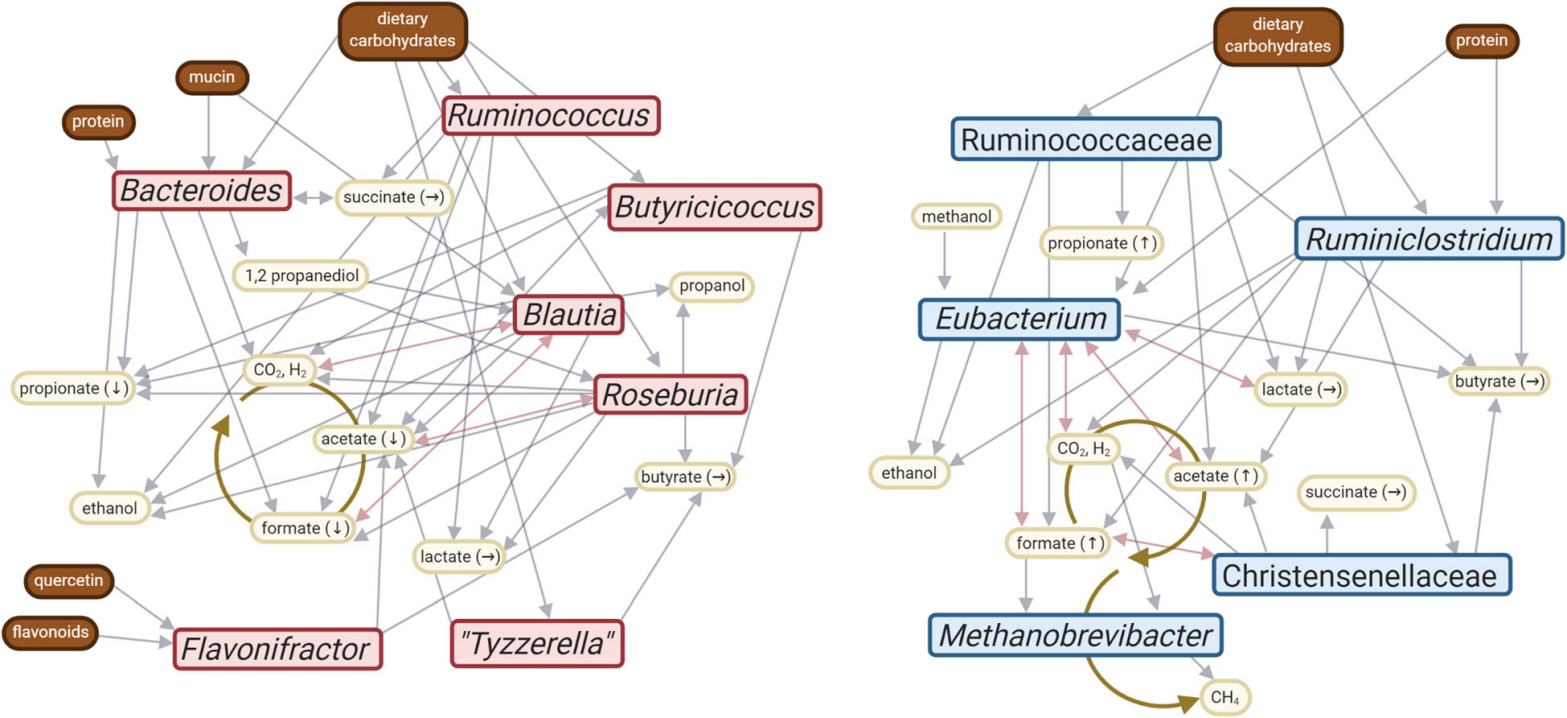
Metabolic network of key-stone taxa in LE (left) and HE (right) microbiomes. Information on the metabolic substrates and products were derived from literature information [52–54]: Lines with arrows, connecting taxa with metabolites, indicate uni-directional (grey) or bi-directional (pink) consumption and/or production. Metabolites measured in stool samples (via metabolomics; this work) are indicated by arrows in brackets following the metabolite name; respective increase (↑) or decrease (↓) of the median by >5% is displayed. For example, a substantially (>5%) increased amount of acetate, propionate and formate was measured in samples from high-methane emitters

Based on literature information available for the keystone taxa [52–54], microbial communities of high- and low-methane emitters are each metabolically highly interwoven. In both cases, degradation of nutrients results in metabolic cycles of short chain fatty acids and CO_2_/H_2_ (Fig. 6). Under LE conditions, these metabolites are trapped in the cycle until they are uptaken by the host or used for microbial biomass production. The conversion of H_2_/CO_2_/formate into methane by *Methanobrevibacter* under HE conditions, however, results in a metabolic ‘dead end’ as methane cannot further be metabolized by gut microbiota or human epithelial cells.

Formate-based methanogenesis is widely distributed amongst human-associated methanogens, as e.g. all *Methanobrevibacter* species detected in a catalogue of 1167 genomes have the capability to use formate for methanogenesis [55]. The ability to consume formate appears to be an important specialization displayed by methanogens in the human gastrointestinal tract and under symbiotic conditions [55]. This hypothesis is supported by the observation that *M. smithii* upregulates formate utilisation gene clusters in syntrophic relationships [56], and methano-archaeal adhesin-like proteins are expressed differently in response to formate, indicating that the physical relationship with bacterial partners changes when different amounts of different metabolites are available [57]. It shall be noted that human-associated *Methanobrevibacter* species are not autotrophs per se but require acetate for biomass production as they generally lack the CODH-ACS complex [58]. Therefore, a higher availability of formate and acetate would support the growth of *M. smithii*.

To characterize the role of the metabolites in more detail and to confirm our assumptions, we performed NMR-based metabolomic analyses of the stool samples (subset, *n*=30). Indeed, we measured an increase in formate concentrations (1.5-fold, based on median concentrations per group) and acetate (1.35-fold) under HE conditions (Fig. 6, Supplementary Table 5; both *p* values > 0.05, *t* test). Propionate was as well increased under HE conditions (1.17-fold), whereas the butyrate, lactate and succinate concentrations remained largely equal (Supplementary Table 5). Formate concentration and methane emissions were significantly correlated (in ppm, Spearman’s rho correlation coefficient 0.491, *p*=0.006). Moreover, formate concentration was significantly correlated with acetate (Spearman rho correlation coefficient 0.785), butyrate (0.416) and propionate (0.447) abundance, whereas no correlations were found for lactate and succinate (0.204 and 0.258, respectively). We can state that the consumption of formate and acetate by *Methanobrevibacter* has large-scale influence on the microbiome composition and functionality, pulling the metabolism strongly towards small carbon compounds in high-methane emitters (see also [51]). In a subsequent step, we were interested in whether subjects’ diet has an influence on these microbial metabolism patterns.

### B12, fat and fibre intake have strong impact on methane microbiomes

A Food Frequency Questionnaire (FFQ) [14] was used to assess the food habits of each participant during the 4 weeks prior to sampling. Overall, the daily intake of 19 nutrients was tracked (Supplementary Table 2). Correlations of all dietary parameters with microbiome and metabolome characteristics is available in Supplementary Table 6 (see also BioEnv plot, Supplementary Fig. 13). *Methanobrevibacter* was negatively correlated with total fat (*r*s=−0.435, *p*=0.016; if not stated otherwise a Spearman’s correlation analysis was performed), saturated fat (*r*s=−0.421, 0.021) and omega-3 fatty acids (*r*s=−0.407, *p*=0.026). Trends indicating a correlation were observed for vitamin B12 intake (*r*s=−0.355, *p*=0.054). Similar negative trends for vitamin B12 (*r*s=−0.465, *p*=0.01) and omega-3 fatty acid (*r*s=−0.349, *p*=0.059) intake were seen when examining the relative abundance of the Christensenellaceae R7 group. Vitamin D intake was negatively correlated with the Christensenellaceae R7 group relative abundances (*r*s=−0.345, *p*=0.062) (Supplementary Table 5).

Within the LE community cluster, an analysis of the genera *Bacteroides*, *Flavonifractor* and the *Ruminococcus gnavus group* revealed a trend with respect to a negative correlation with dietary fibre intake (*r*s=−0.379, *p*=0.039; *r*s=−0.517, *p*=0.003 and *r*s=−0.382, *p*=0.037, respectively). The relative abundance of *Blautia* positively correlated with vitamin B12 levels (*r*s=0.505, *p*=0.004) and protein intake (rs=0.422, *p*=0.020). Vegetarianism correlated with different dietary compound intake, namely, vitamin C and sugar intake was positively correlated (*r*s=0.490, *p*=0.006 and *r*s=0.441, *p*=0.015, respectively), whereas food diversity and vitamin B12 levels (*r*s=−0.473, *p*=0.008 and *r*s=−0.449, *p*=0.013, respectively) were negatively correlated with vegetarianism (Supplementary Table 5).

Based on dietary information, vitamin B12 (cobalamin) appeared to be an important modulatory factor. The key-role of vitamin B12 was further supported by the significant negative correlation of formate concentration in the fecal samples and vitamin B12 uptake (*p*=0.038, *R*=−0.380).

Vitamin B12 (cobalamin) is an important micronutrient, as it is involved in a number of homeostatic functions of host and microbiome. The host absorbs cobalamin solely in the small intestine, not disturbing the metabolic cycle of microbial cobalamin-producers (approx. 25% of all gut bacteria) and –consumers (particularly *Bacteroides*) in the large intestine [59]. Following our observations on the negative correlation of B12 and methanogenesis, indeed, functions involved in B12 binding and transportation were significantly increased in LE metagenomes (B12-binding component *BtuF*, *p*=0.004, *t* test; Supplementary dataset 3).

Notably, formate and vitamin B12 (cobalamin) metabolism are closely connected also in humans. Cobalamin deficiency was associated with increased formate concentrations in urine and plasma (in rats, [60]), due to the so-called methyl-folate trap [61–63]. Under these conditions, the cytosolic folate accumulates as 5-methyl-THF (thus reducing the concentration of THF), which impedes the incorporation of formate into the folate pool, and results in formate accumulation. In general, replenishing the THF pool also involves ALDH1L1 (10-formyltetrahydrofolate dehydrogenase), an enzyme involved in formate oxidation, which converts 10-formyl-THF to THF and CO_2_. Notably, an association between the Christensenellaceae/*Methanobrevibacter* abundance and the abundance of a certain SNP (rs2276731) in the ALDH1L1 gene was observed when genetic correlations with microbiome profiles were analysed in a large UK twin study [64]. SNP rs2276731 is characterized by a nucleotide exchange towards C (instead of G, T) in approx. 17% of the population [65]. This ratio is in high agreement with the percentage of methane producers observed in our (15%) and other studies [9].

As *Methanobrevibacter* appears to be able to grow independently from cobalamin availability [56], it could benefit from the increased formate (and acetate) concentrations in the GIT, without being influenced by possible vitamin B12 shortage.

### Individual diet-adapted flux balance analysis confirms the vitamin-independent, maximal breakdown of fibre to C1 metabolites under HE conditions

In order to draw an analogy of dietary information and the identified key taxa, we performed a flux balance analysis with MICOM [47]. To optimize this approach to our scientific question, we included the individual dietary information obtained from the donors in our model (Supplementary Dataset 7). The community models were based on the AGORA 1.03 genus model [48]. Growth simulations resulted in information on growth rates, growth niches, metabolite consumptions and phenotype associated fluxes (Supplementary Dataset 5 and 6).

**Fig. 7:**
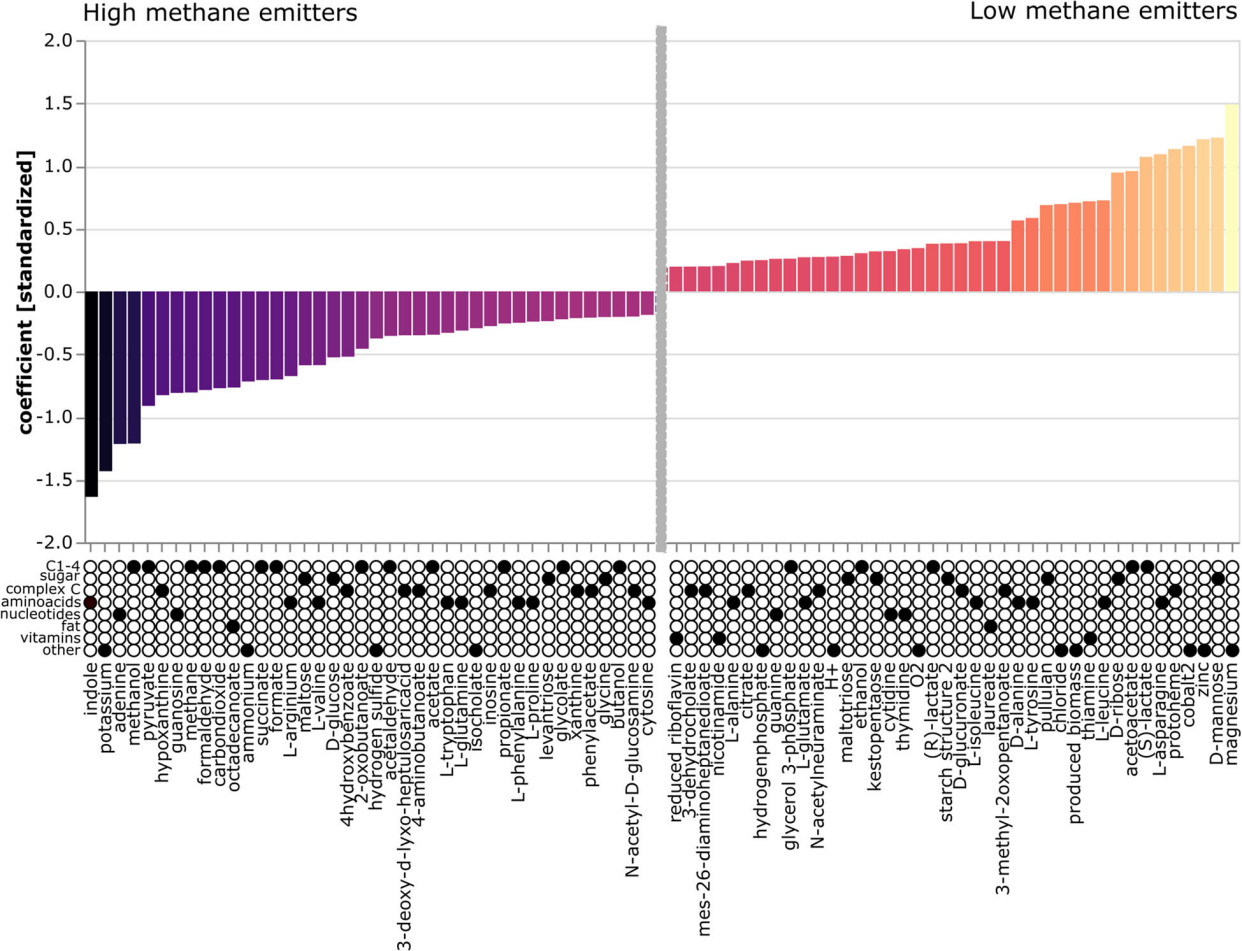
MICOM model-based flux balance analysis of keystone taxa. The 40 most predictive production fluxes (metabolites) are shown for high-methane emitters on the left and low-methane emitters to the right using L1 penalized logistic regression. Black dots underneath are used to display the categorization of each metabolite into different types of metabolites. The analysis was based on 16S rRNA gene amplicon data of the matched cohort (*n*=30) and the identified keystone taxa

The results of the analysis performed on previously identified keystone taxa confirmed a significant association between the HE conditions and an increased flux of C1 metabolites, such as methanol, formaldehyde, carbon dioxide and formate (Fig. 7), as well as acetate and propionate. LE conditions were associated with d-mannose, lactate, ribose levels and overall a greater complexity of organic molecules. Notably, the hydrogen flux was only minimally associated with HE (−0.021595761). Fluxes in vitamin compounds (nicotinamide, riboflavin, thiamine, pyridoxin, menaquinone 8) were strongly associated with the LE conditions. The outcome of the modelling approach strongly confirmed our above-made observations based on functional microbiome and metabolome analyses and indicated the further involvement of other components, such as methanol or indole, which require further investigation.

## Discussion

In this study, we analyzed the underlying principle of human methane emission. We were able to show the following:

i) High-methane emission is correlated with a more complex microbiome in the GIT
ii) The microbial community composition and function differs significantly between high- and low-methane emitters and is pronounced in specific archaeal and bacterial key-taxa
iii) *Methanobrevibacter smithii*, whose abundance is increased by a factor of 1,000 under HE conditions, pulls microbiome function towards acetate and formate production
iv) Dietary habits, including low B12 uptake, support optimal gastrointestinal conditions for a complete and efficient break-down of fibres to C1 compounds with a low need for vitamins.

The abundance of *Methanobrevibacter* was strongly correlated with a core group of keystone species, including various Ruminococcaceae and Christensenellaceae (see also [66]). The interplay between *Methanobrevibacter* and Christensenellaceae is of great interest, as this syntrophic partnership has been associated with a lean phenotype [67] and a reduced gain of fat tissue [68, 69] in earlier publications. Notably, both taxa are considered to be highly inheritable [53, 67]. In co-culturing studies, the methanogenic partner shifted the *Christensenella minuta* metabolism, probably due to its potent hydrogen consumption, toward acetate production rather than toward butyrate production, leading to increased H_2_ and CO_2_ production [51, 67]. Although this observation would indicate a bilateral syntrophic relationship of both microorganisms, we observed in our study that both partners were unevenly affected by LE and HE conditions: Christensenellaceae were present in both communities (2% in LE), and signatures increased only three-fold towards HE conditions, whereas *Methanobrevibacter* signatures increased 1,000-fold, probably indicating a more complex underlying principle. Indeed, we could not identify any dietary-derived compound which had a direct, significantly stimulating or inhibiting effect on the Christensenellaceae population.

The complexity of ingested saccharides is an important modulator for the composition and functionality of a gastrointestinal microbiome, and an interesting link between cellulose degradation and methane emission was observed by other researchers. Chassared et al. (2010) described that dominant cellulose degraders isolated from non-methane-excreting subjects are mainly affiliated with Bacteroidetes, while they are predominantly represented by Firmicutes in methane-excreting individuals [70]. In our study, we also identified *Bacteroides* (Bacteroidetes) and *Roseburia* (Firmicutes), as well as Christensenellaceae, *Ruminiclostridium* and *Ruminococcaceae* (Firmicutes), as important key taxa in LE and high-methane-emitting subjects, respectively. Notably, *Bacteroides* (which was shown to be significantly negatively correlated with dietary fibres in our study) and *Roseburia*, unlike *Ruminococcus* sp., are not able to digest e.g. microcrystalline cellulose [70–72]. This indicates that the type of dietary fibre has a potential modulating impact on methane production.

The negative correlations observed for fat intake and methanogen abundance are highly congruent with previous observations made in ruminants, where an increased fat (oil) concentration in the diet led to a reduced enteric methane production of up to 36% ([73] and references therein). It is considered that dietary fat affects methane production in rumen because it reduces the hydrogen accumulation through fatty acid biohydrogenation, leading to the conversion of unsaturated fatty acids to saturated fatty acids, reducing the intake of fermentable organic matter and fibre digestion [73].

### Study limitations

The findings of this study are based on a homogenous study group (e.g. neither elderly persons nor children were recruited), and thus, no general conclusions can be drawn regarding the impact of methanogen presence on aging, health status or obesity. Future studies are needed to collect data from more variable study groups with more individuals and to examine the longitudinal dynamics of the HE microbiome in more detail in terms of its correlation with additional parameters (e.g. blood metabolites). Although we were able to partially confirm the information derived from 16S rRNA gene-based metabolic flux analysis, other identified metabolites require as well confirmation via metabolomics or other means. One of these examples is indole, for which a substantial role was proposed in HEs (Fig. 7). Indoles are usually derived from gut microbial conversion of tryptophan and have a variety of important functions, including host defense and fortifying the gut barrier. Moreover, indoles are important, dose-dependent signaling molecules for bacteria, with effect on motility, biofilm formation, antibiotic resistances and virulence [52, 74]. As this might have large physiological and maybe medical effects on the host, this aspect certainly warrants additional studies in future.

## Conclusions

High-methane baseline emission in breath mirrors a complex situation of the human physiology, including vitamin B12 shortage and increased formate levels in the GIT. Higher formate levels were earlier, and independently from methane breath analyses, correlated with positive foetal development, T cell activation, a lean phenotype, and cardiovascular function [75]. Thus, the correlation of high-methane emission and formate concentration warrants future research. Moreover, as we revealed the impact of dietary fibre, vitamin and fat uptake on methanogenic activity, dietary modulations (e.g. vitamin B12 supplementation) could be used for the mitigation of methane-associated disorders, such as constipation. Our study and its results emphasize the importance of archaeome activity in the human body. This activity serves as an important mirror, modulator and regulator of the microbiome and overall body processes.

## Supplementary Information

**Additional file 1: Supplementary Figures. Supplementary Figure 1.** Microbiome profiles and differences in abundances of specific taxa in HEs compared to LEs based on the “universal” approach (16S rRNA gene sequencing). A. Whole study cohort (*n*=100). B. Matched study subset (*n*=30). AI/BI. PCoA plots (RSV based); AII/BII. ANOVA analysis at phylum level and AIII/BIII at genus level on the 100 most abundant taxa. AIV-VII/BIV-VII. Relative abundances of individual genera. **Supplementary Figure 2.** Significant positive and negative correlation of specific taxa with emitted methane concentrations based on “universal” approach 16S rRNA gene sequencing, Spearman-based regression analysis. A. Whole study cohort (*n*=100). B. matched study subset only (*n*=30). I-V. Significant positive correlation with emitted methane. VI-X. Significant negative correlation with emitted methane. (100 most abundant genera; Spearman); r=Spearman’s rho correlation coefficient (rs). **Supplementary Figure 3.** Co-correlation network of taxa associated with HE and LE based on “universal” approach 16S rRNA gene sequencing and Spearman’s rho. Networks showing connections of the 100 most abundant genera of A. the whole study cohort (*n*=100), B. our matched study subset (n=30), C. HE only (n=15) and D. LE only (*n*=15). Taxa highlighted in red and blue were shown to be most significantly different in LEfSe and ANOVA analysis. **Supplementary Figure 4.** High methane emitters showed higher absolute abundances of methanogens compared to low emitters (n=100). Bacterial absolute abundances was overall similar. For three samples, data of bacteria was not obtained. **Supplementary Figure 5.** Differences in alpha and beta diversity based on the “universal” approach of 16S rRNA gene sequencing between high (HE) and low methane emitters (LE). Profile of the study subset (n=30). A.I. The Shannon diversity index of revealed significant differences in alpha diversity between both groups (RSV based; ANOVA); A.II. Microbial community clustered significantly different in the RDA plot (RSV based). B.I. LeFSe analysis of the 100 most abundant phyla and B.II.-III. Relative abundance of selected phyla shown in ANOVA plots. C.I. LeFSe analysis of the 100 most abundant genera and C.II.-III. Relative abundance of selected phyla shown in ANOVA plots. **Supplementary Figure 6.** Bubble plot overview on subsystems at the highest (I.) and at functional level (II.) based on shotgun metagenome analysis. In II., the 50 most abundant features are shown.; n=30. **Supplementary Figure 7**. Relative abundance of the most significantly different subsystems of HEs compared to LEs shown in ANOVA plots based on shotgun metagenome analysis (Subsystems). I. At highest subsystem level (level 1) and II. level3. (100 most features; n=30). **Supplementary Figure 8.** Bubble plots of gut microbiome of HEs and LEs based on shotgun metagenome (RefSeq). I. **Supplementary Figure 9.** Significant differences were also observed at species level based on shotgun metagenome analysis (RefSeq). I. Bubble plot of the 50 most abundant taxa. II. LefSe analysis and III. ANOVA plot of 100 most abundant taxa. (n=30). **Supplementary Figure 10.** Microbial community differs significantly with respect to methane production based on shotgun metagenome analysis (RefSeq). I. LEfSe analysis and II. ANOVA plot at superkingdom level. III. PCoA plot at RSV level. IV. ANOVA plot showing significant differences at phylum (100 most abundant) and V. genus level (50 most abundant taxa). (n=30). **Supplementary Figure 11.** Diversity and composition of the archaeal community as detected in HE and LE samples based on shotgun metagenomic analyses (RefSeq). I Alpha diversity based on Shannon index, II. RDA plot, III. PCoA plot, IV: LEfSe analysis on genus level. **Supplementary Figure 12.** Archaeal network in LE and HE (blue) based on shotgun metagenomics information (RefSeq). **Supplementary Figure 13.** Correlations with dietary intake. BIOENV analysis showing explanatory variables triggering the microbial communities of HEs (blue) and LEs (red) based on Euclidean distances that were superimposed on a Non-metric multidimensional scaling (NMDS) plot derived from Bray-Curtis dissimilarities of HE and LE samples (stress:0.1939). *Methanobrevibacter* read counts were included as a variable for better orientation.

**Additional file 2: Supplementary Tables. Supplementary Table 1.** Characteristics of all participants (*n*=100). **Supplementary Table 2.** Characteristics of the matched subset (*n*=30). **Supplementary Table 3.** Key stone taxa of high and low methane emitters (*n*=30). Identified key taxa based on LEfSe analysis of the 600 most abundant genera/RSVs. Numbers in column 2 refer to Fig. 5. **Supplementary Table 4.** High quality dereplicated key MAGs including quality and replication estimates as well as taxonomic classification according to GTDB. **Supplementary Table 5.** Metabolite concentrations in high and low methane emitters (*n*=30). **Supplementary Table 6.** Correlations of different parameters (general, keystone taxa, metabolites and diet) of this study. **Supplementary Table 7.** qPCR data of all study participants. **Supplementary Table 8.** Sequence information and analysis statistics by MG-Rast.

**Additional file 3: Supplementary Items 6-13. Supplementary Item 6.** Heatmap of amino acid flux predictions according to MICOM (universal primer: 515F-806R; *n*=30). **Supplementary Item 7.** Heatmap of C1-C4 flux predictions according to MICOM (universal primer: 515F-806R; *n*=30). **Supplementary Item 8.** Heatmap of complex compound flux predictions according to MICOM (universal primer: 515F-806R; *n*=30). **Supplementary Item 9.** Heatmap of fat flux predictions according to MICOM (universal primer: 515F-806R; *n*=30). **Supplementary Item 10.** Heatmap of nucleotide flux predictions according to MICOM (universal primer: 515F-806R; *n*=30). **Supplementary Item 11.** Heatmap of other metabolite flux predictions according to MICOM (universal primer: 515F-806R; *n*=30). **Supplementary Item 12.** Heatmap of sugar flux predictions according to MICOM (universal primer: 515F-806R; *n*=30). **Supplementary Item 13.** Heatmap of vitamine flux predictions according to MICOM (universal primer: 515F-806R; *n*=30).

**Additional file 4: Supplementary Datasets. Supplementary Dataset 1.** Feature table amplicon data of universal approach (universal primer: 515F-806R; *n*=100). **Supplementary Dataset 2.** Feature table amplicon data of archaeal approach (nested PCR: 344F-1041R, 519F-806R; (n=100). **Supplementary Dataset 3.** Feature table metagenomic data showing functional gene information (SEED; *n*=30). **Supplementary Dataset 4.** Feature table metagenomic data showing taxonomic information (RefSeq; *n*=30). **Supplementary Dataset 5**. MICOM growth rate predictions (universal primer: 515F-806R; *n*=30). **Supplementary Dataset 6.** MICOM metabolite flux predictions (universal primer: 515F-806R; *n*=30). **Supplementary Dataset 7.** Adapted per sample diet model for MICOM (*n*=30).

**Additional file 5: Supplementary Item 1.** Krona chart based on amplicon data (universal, *n*=100).

**Additional file 6: Supplementary Item 2.** Krona chart based on amplicon data (archaea, *n*=100).

**Additional file 7: Supplementary Item 3.** Krona chart based on metagenomic data (SEED, *n*=30).

**Additional file 8: Supplementary Item 4.** Krona chart based on metagenomic data (RefSeq, archaea and bacteria only, *n*=30).

**Additional file 9: Supplementary Item 5.** Krona chart based on metagenomic data (RefSeq, archaea only, *n*=30).

**Additional file 10.**

## Data Availability

Raw sequencing data obtained from amplicon-based sequencing and metagenomics sequencing data (technical sequences including adaptor sequences, linker sequences and barcode sequences as well as human reads were removed) used in this paper can be found in the European Nucleotide Archive (ENA): PRJEB41867. Supplementary Datasets (after decontam and removal of features with zero and one reads) and all Supplementary Figures, Tables and Items were additionally deposited on Mendeley at 10.17632/hjj3tx7n84.1.
