# Supplementary material for: Reduced B12 uptake and increased gastrointestinal formate are associated with archaeome-mediated breath methane emission in humans
Source: Microbiome. 2021 Sep 24;9:193. doi: 10.1186/s40168-021-01130-w (PMC8464155; doi:10.1186/s40168-021-01130-w)
Supplement: Supplementary file 2 — Additional file 1: Supplementary Figures. Supplementary Figure 1. Microbiome profiles and differences in abundances of specific taxa in HEs compared to LEs based on the “universal” approach (16S rRNA gene sequencing). A. Whole study cohort (n=100). B. Matched study subset (n=30). AI/BI. PCoA plots (RSV based); AII/BII. ANOVA analysis at phylum level and AIII/BIII at genus level on the 100 most abundant taxa. AIV-VII/BIV-VII. Relative abundances of individual genera. Supplementary Figure 2. Significant positive and negative correlation of specific taxa with emitted methane concentrations based on “universal” approach 16S rRNA gene sequencing, Spearman-based regression analysis. A. Whole study cohort (n=100). B. matched study subset only (n=30). I-V. Significant positive correlation with emitted methane. VI-X. Significant negative correlation with emitted methane. (100 most abundant genera; Spearman); r=Spearman’s rho correlation coefficient (rs). Supplementary Figure 3. Co-correlation network of taxa associated with HE and LE based on “universal” approach 16S rRNA gene sequencing and Spearman’s rho. Networks showing connections of the 100 most abundant genera of A. the whole study cohort (n=100), B. our matched study subset (n=30), C. HE only (n=15) and D. LE only (n=15). Taxa highlighted in red and blue were shown to be most significantly different in LEfSe and ANOVA analysis. Supplementary Figure 4. High methane emitters showed higher absolute abundances of methanogens compared to low emitters (n=100). Bacterial absolute abundances was overall similar. For three samples, data of bacteria was not obtained. Supplementary Figure 5. Differences in alpha and beta diversity based on the “universal” approach of 16S rRNA gene sequencing between high (HE) and low methane emitters (LE). Profile of the study subset (n=30). A.I. The Shannon diversity index of revealed significant differences in alpha diversity between both groups (RSV based; ANOVA); A.II. Microbial community c [file 40168_2021_1130_MOESM2_ESM.pdf]

Supplementary Figures

Reduced B12 uptake and increased gastrointestinal formate drive archaeome-mediated breath methane emission in humans

Short title: B12 shortage, fibre and formate drive breath methane emission

Christina Kumpitsch,[1] Florian Ph. S. Fischmeister,[2,3] Alexander Mahnert,[1] Sonja Lackner,[4] Marilena Wilding,[2] Corina Sturm,[2] Anna Springer,[5] Tobias Madl,[5,6] Sandra Holasek,[4] Christoph Högenauer,[7] Ivan Berg,[8] Veronika Schoepf,[3] Christine Moissl-Eichinger[1,6*]

[1] Diagnostic and Research Institute of Hygiene, Microbiology and Environmental Medicine, Medical University of Graz, Graz 8010, Austria,

[2] Department of Psychology, University of Graz, Graz 8010, Austria,

[3] Department of Biomedical Imaging and Image-guided Therapy, Medical University of Vienna, Vienna 1090, Austria,

[4] Division of Immunology and Pathophysiology, Medical University of Graz, Graz 8010, Austria,

[5] Gottfried Schatz Research Center for Cell Signaling, Metabolism and Aging, Molecular Biology & Biochemistry, Medical University of Graz, Graz 8010, Austria,

[6] BioTechMed, Graz, Graz 8010, Austria,

[7] Division of Gastroenterology and Hepatology, Medical University of Graz, Graz, Austria,

[8] Institute for Molecular Microbiology and Biotechnology, University of Münster, Münster, Germany.

* Correspondence: Christine Moissl-Eichinger, Medical University of Graz, Diagnostic and Research Institute of Hygiene, Microbiology and Environmental Medicine, Neue Stiftingtalstraße 6, 8010 Graz, Austria, christine.moissl-eichinger@medunigraz.at

Keywords: archaeome, microbiome, methanogens, methane, gut, gastrointestinal tract, metabolome, metagenome, *Methanobrevibacter*, Christensenellaceae

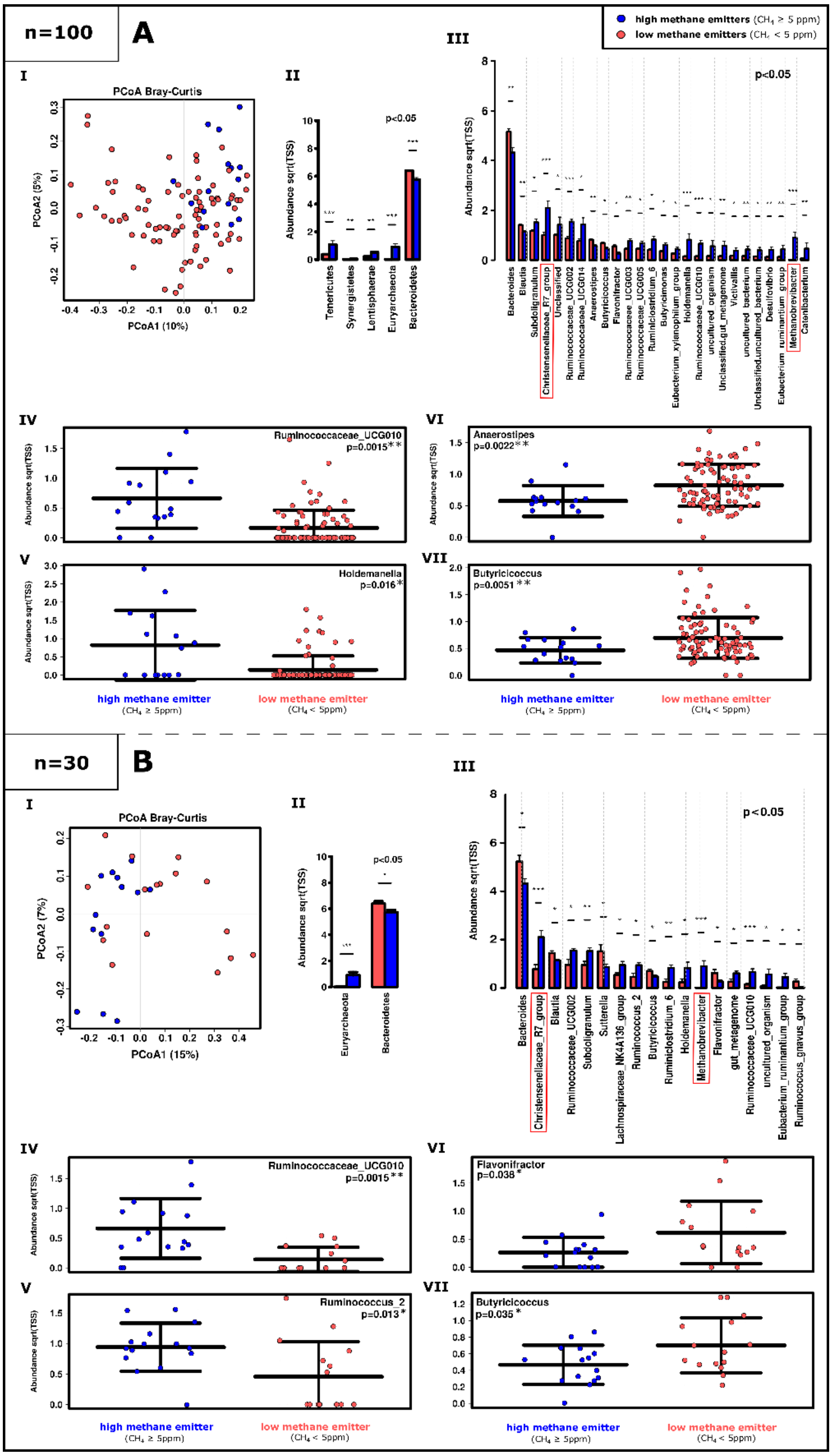

Supplementary Figure 1. Microbiome profiles and differences in abundances of specific taxa in HEs compared to LEs based on the "universal" approach (16S rRNA gene sequencing). **A.** Whole study cohort (n=100). **B.** Matched study subset (n=30). **AI/BI.** PCoA plots (RSV based); **AII/BII.** ANOVA analysis at phylum level and **AIII/BIII** at genus level on the 100 most abundant taxa. **AIV-VII/BIV-VII.** Relative abundances of individual genera.

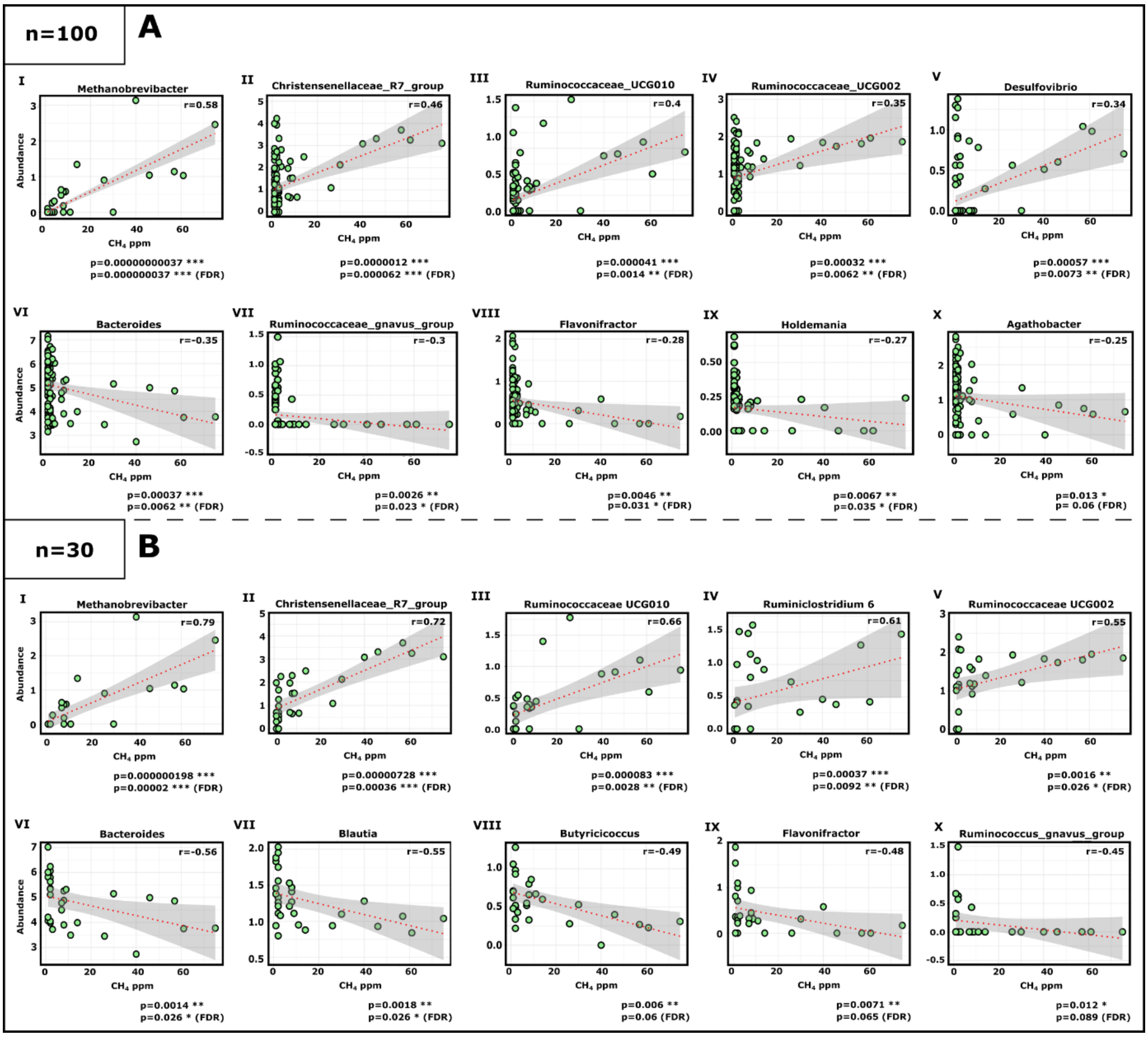

Supplementary Figure 2. Significant positive and negative correlation of specific taxa with emitted methane concentrations based on "universal" approach 16S rRNA gene sequencing, Spearman-based regression analysis. A. Whole study cohort (n=100). **B.** matched study subset only (n=30). **I-V.** Significant positive correlation with emitted methane. **VI-X.** Significant negative correlation with emitted methane. (100 most abundant genera; Spearman); r=Spearman's rho correlation coefficient (*r*s)

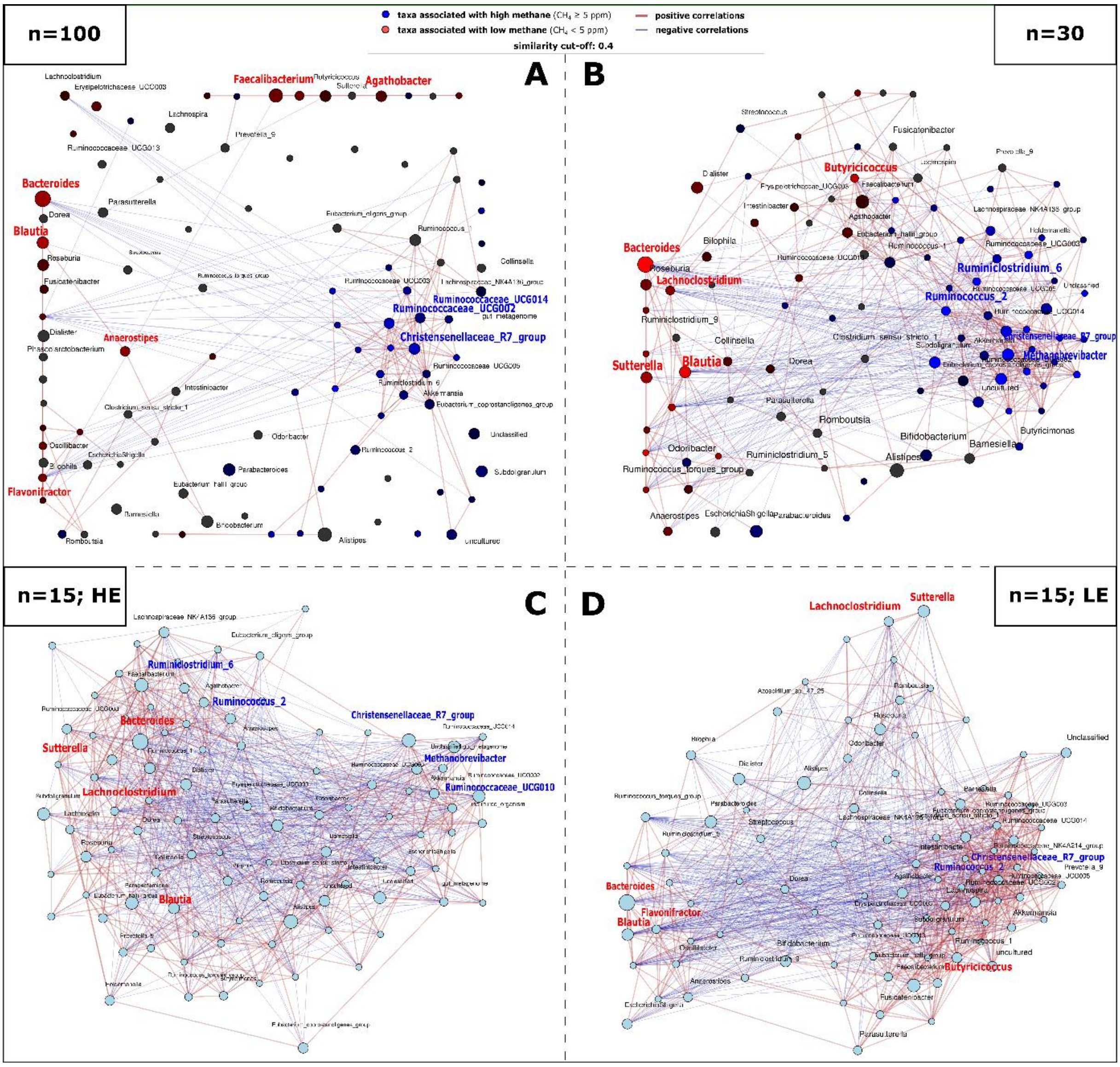

Supplementary Figure 3. Co-correlation network of taxa associated with HE and LE based on "universal" approach 16S rRNA gene sequencing and Spearman's rho. Networks showing connections of the 100 most abundant genera of **A.** the whole study cohort (n=100), **B.** our matched study subset (n=30), **C.** HE only (n=15) and **D.** LE only (n=15). Taxa highlighted in red and blue were shown to be most significantly different in LEfSe and ANOVA analysis.

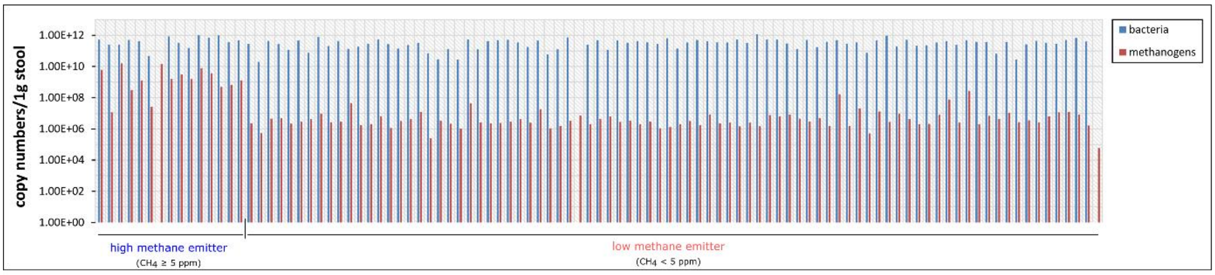

Supplementary Figure 4. High methane emitters showed higher absolute abundances of methanogens compared to low emitters (n=100). Bacterial absolute abundances was overall similar. For three samples, data of bacteria was not obtained.

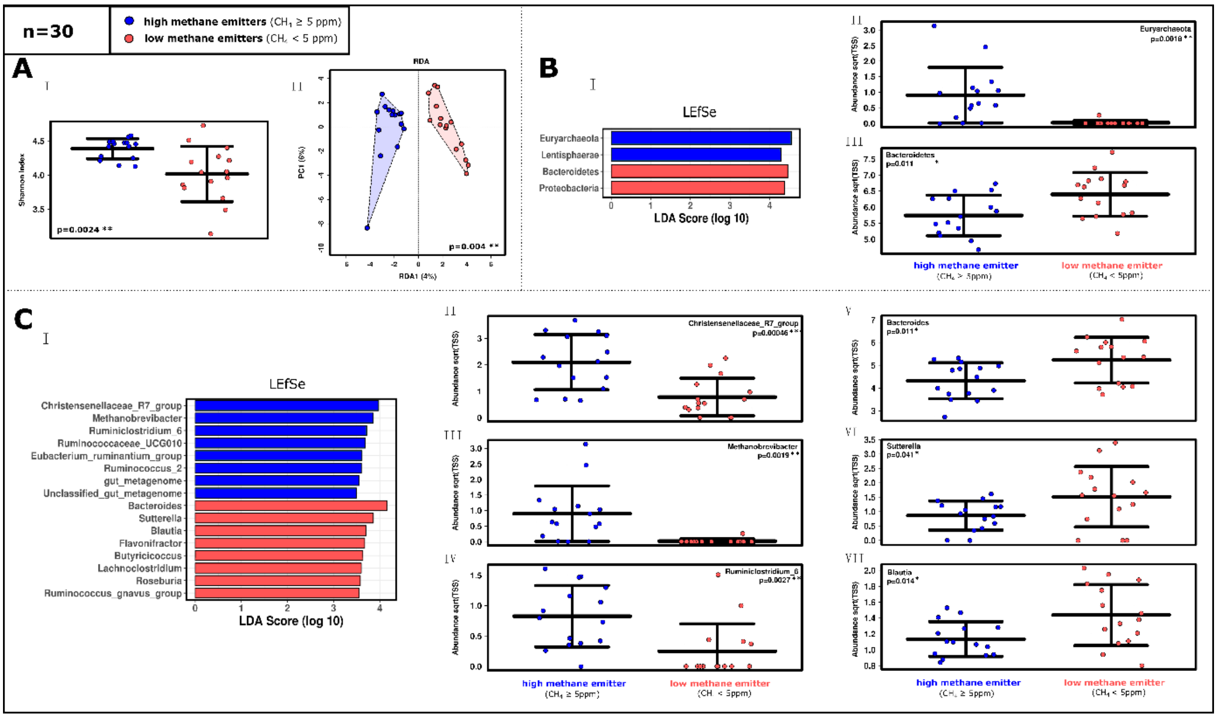

Supplementary Figure 5. Differences in alpha and beta diversity based on the "universal" approach of 16S rRNA gene sequencing between high (HE) and low methane emitters (LE). Profile of the study subset (n=30). **A.I.** The Shannon diversity index of revealed significant differences in alpha diversity between both groups (RSV based; ANOVA); **A.II.** Microbial community clustered significantly different in the RDA plot (RSV based). **B.I.** LeFSe analysis of the 100 most abundant phyla and **B.II.-III.** Relative abundance of selected phyla shown in ANOVA plots. **C.I.** LeFSe analysis of the 100 most abundant genera and **C.II.-III.** Relative abundance of selected phyla shown in ANOVA plots.

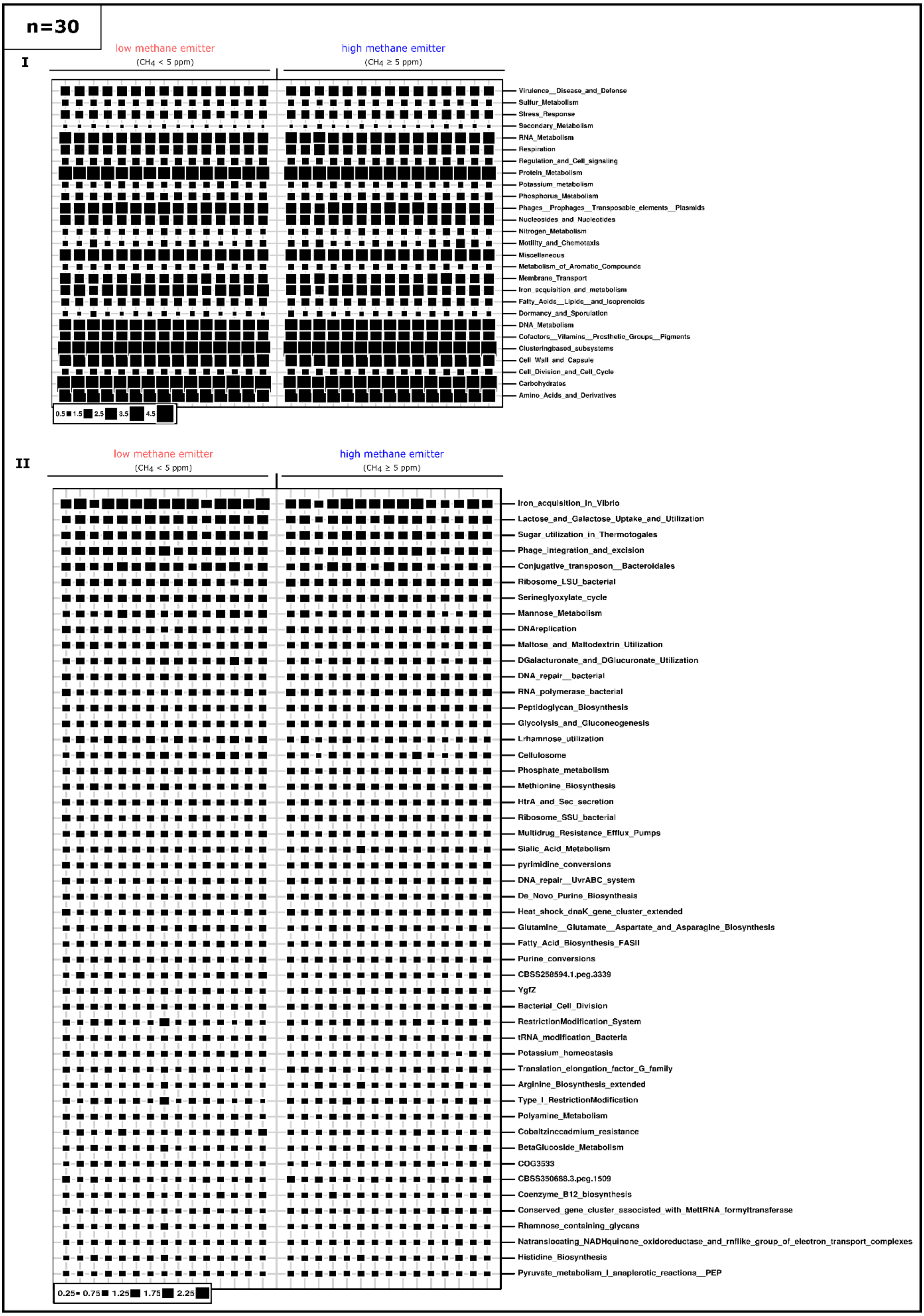

Supplementary Figure 6. Bubble plot overview on subsystems at the highest (I.) and at functional level (II.) based on shotgun metagenome analysis. In II., the 50 most abundant features are shown.; n=30.

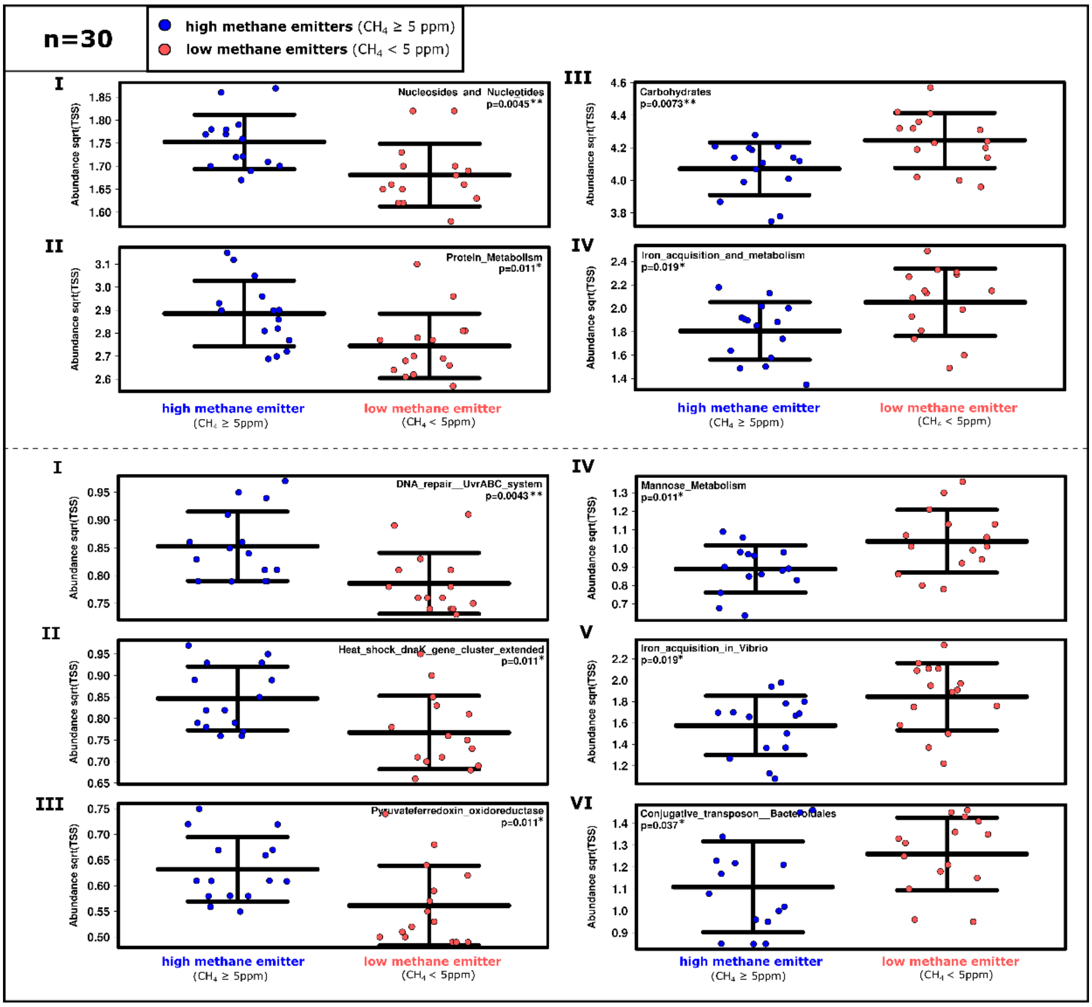

Supplementary Figure 7. Relative abundance of the most significantly different subsystems of HEs compared to LEs shown in ANOVA plots based on shotgun metagenome analysis (Subsystems). I. At highest subsystem level (level 1) and II. level3. (100 most features; n=30)

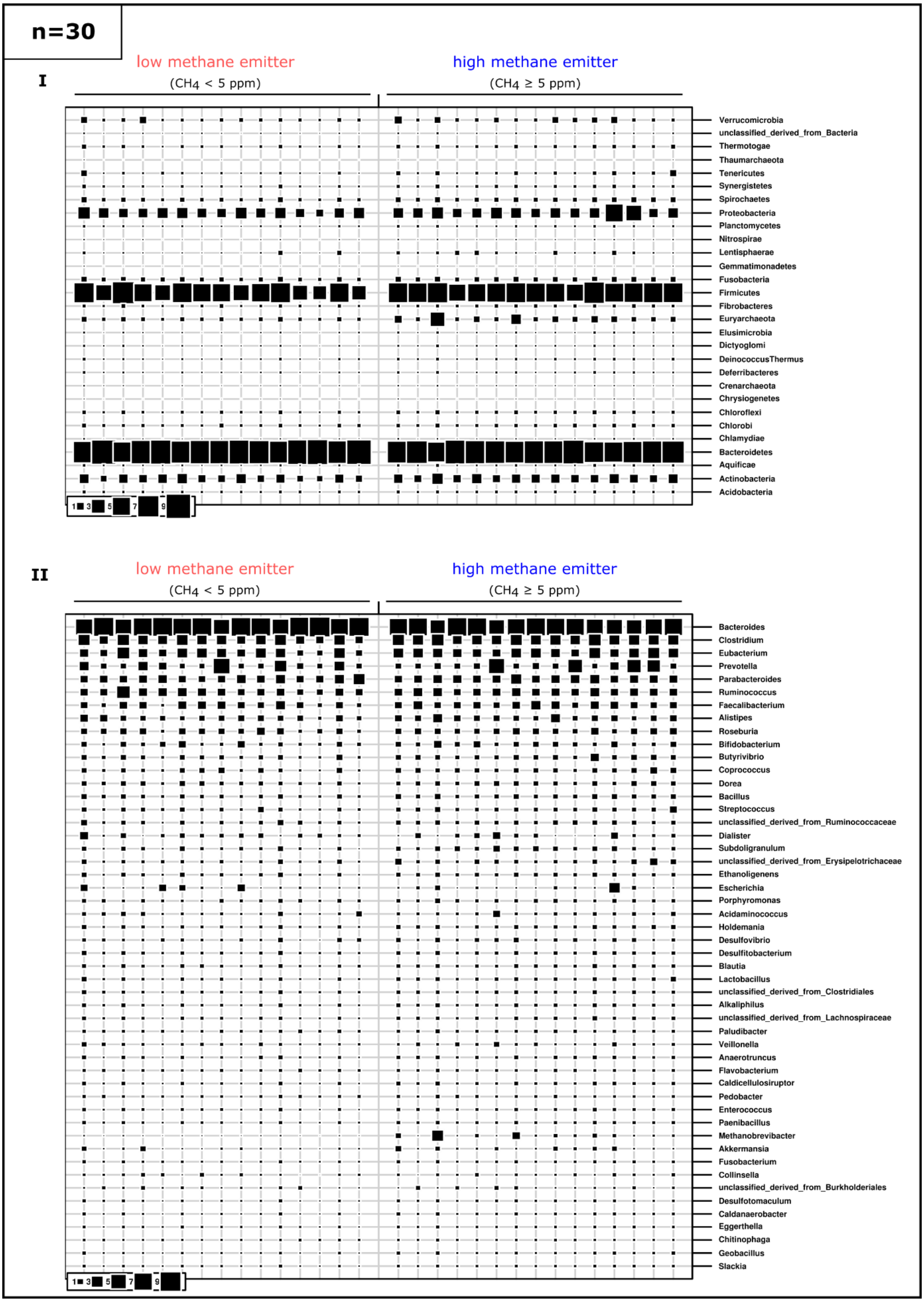

Supplementary Figure 8. Bubble plots of gut microbiome of HEs and LEs based on shotgun metagenome (RefSeq). I. visualized at phylum level and **II.** genus level. (50 most abundant taxa; n=30)

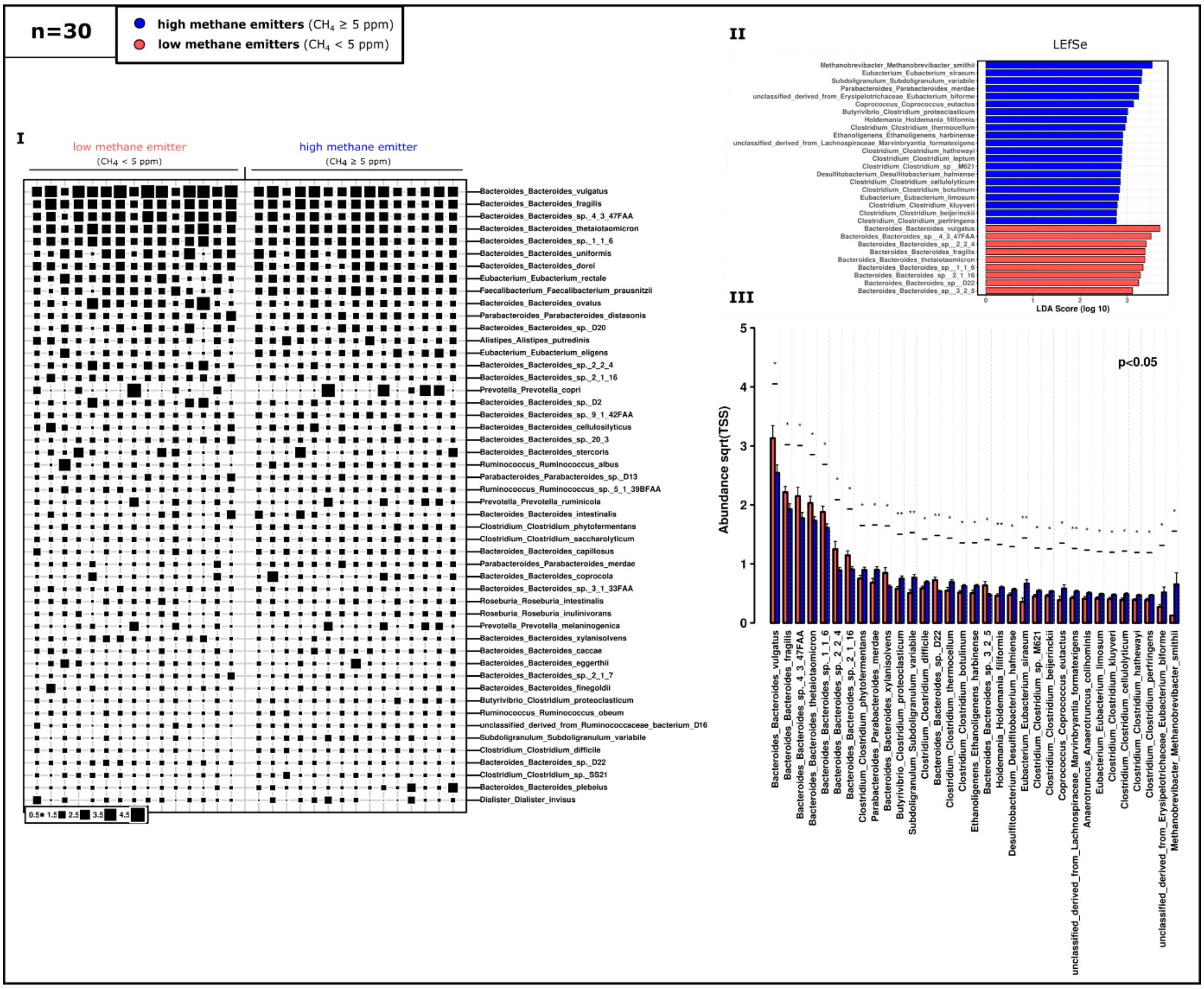

Supplementary Figure 9. Significant differences were also observed at species level based on shotgun metagenome analysis (RefSeq). I. Bubble plot of the 50 most abundant taxa. II. LefSe analysis and III. ANOVA plot of 100 most abundant taxa. (n=30)

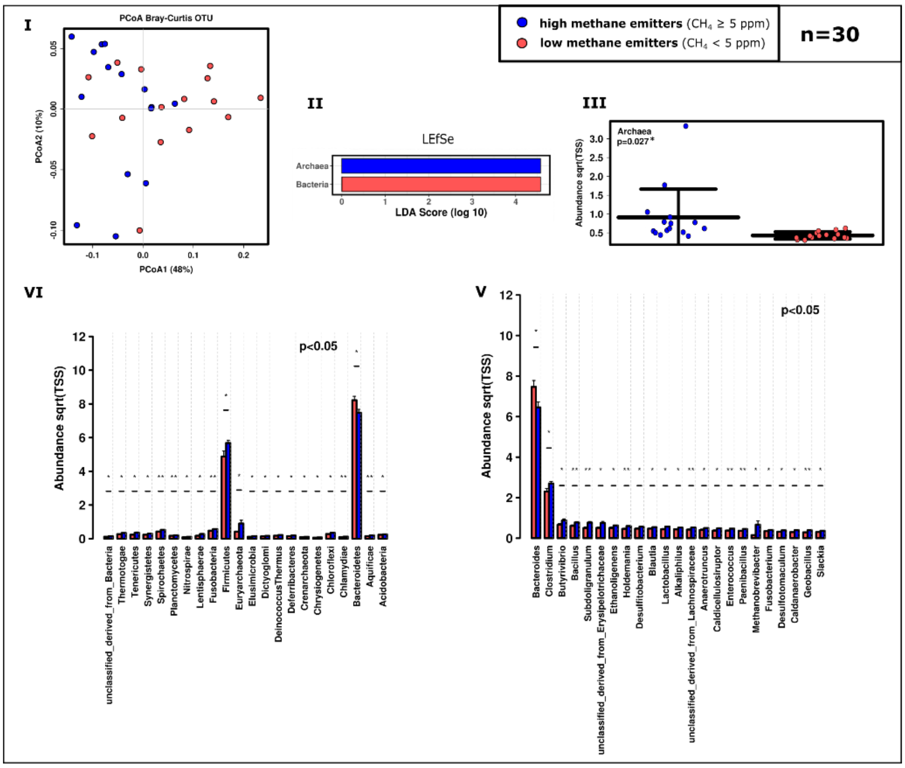

Supplementary Figure 10. Microbial community differs significantly with respect to methane production based on shotgun metagenome analysis (RefSeq). I. LEfSe analysis and **II.** ANOVA plot at superkingdom level. **III.** PCoA plot at RSV level. **IV.** ANOVA plot showing significant differences at phylum (100 most abundant) and **V.** genus level (50 most abundant taxa). (n=30)

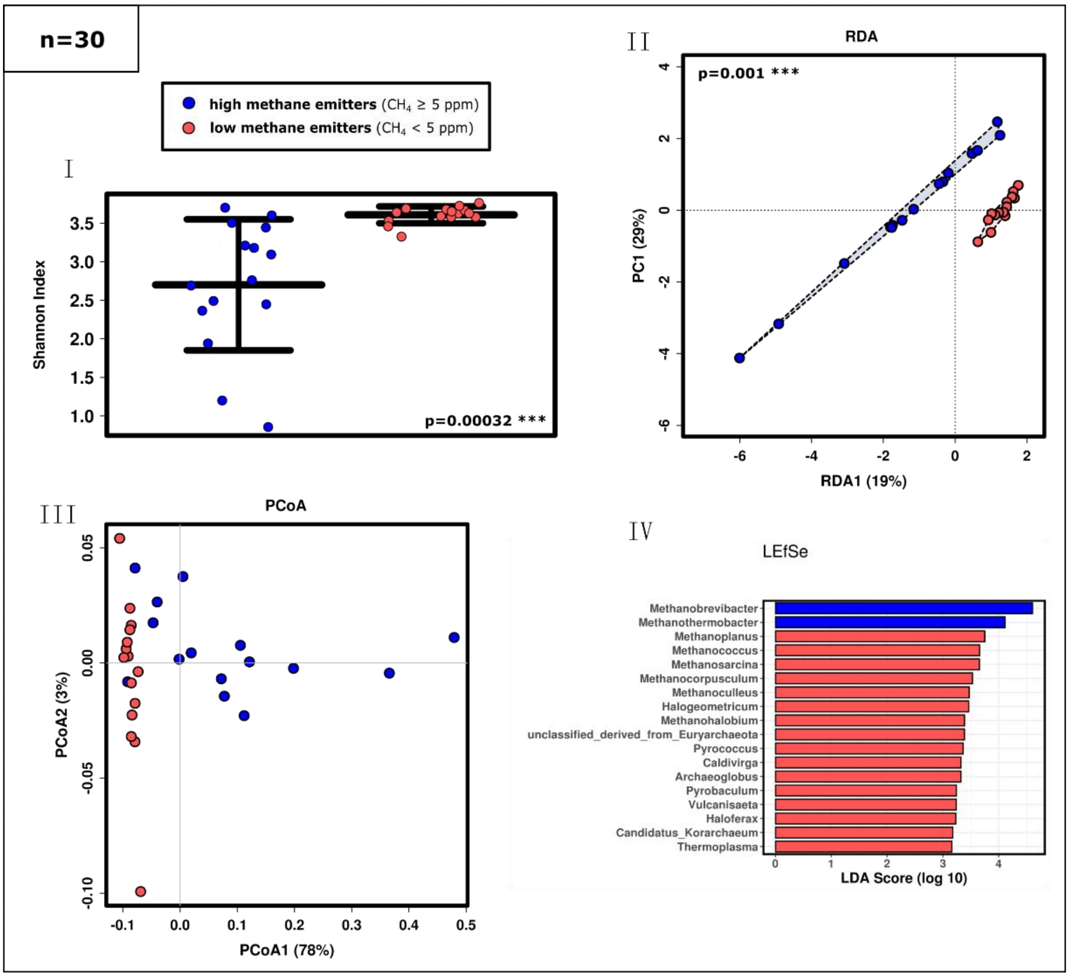

Supplementary Figure 11. Diversity and composition of the archaeal community as detected in HE and LE samples based on shotgun metagenomic analyses (RefSeq). I Alpha diversity based on Shannon index**, II.** RDA plot**, III.** PCoA plot**, IV:** LEfSe analysis on genus level**.**

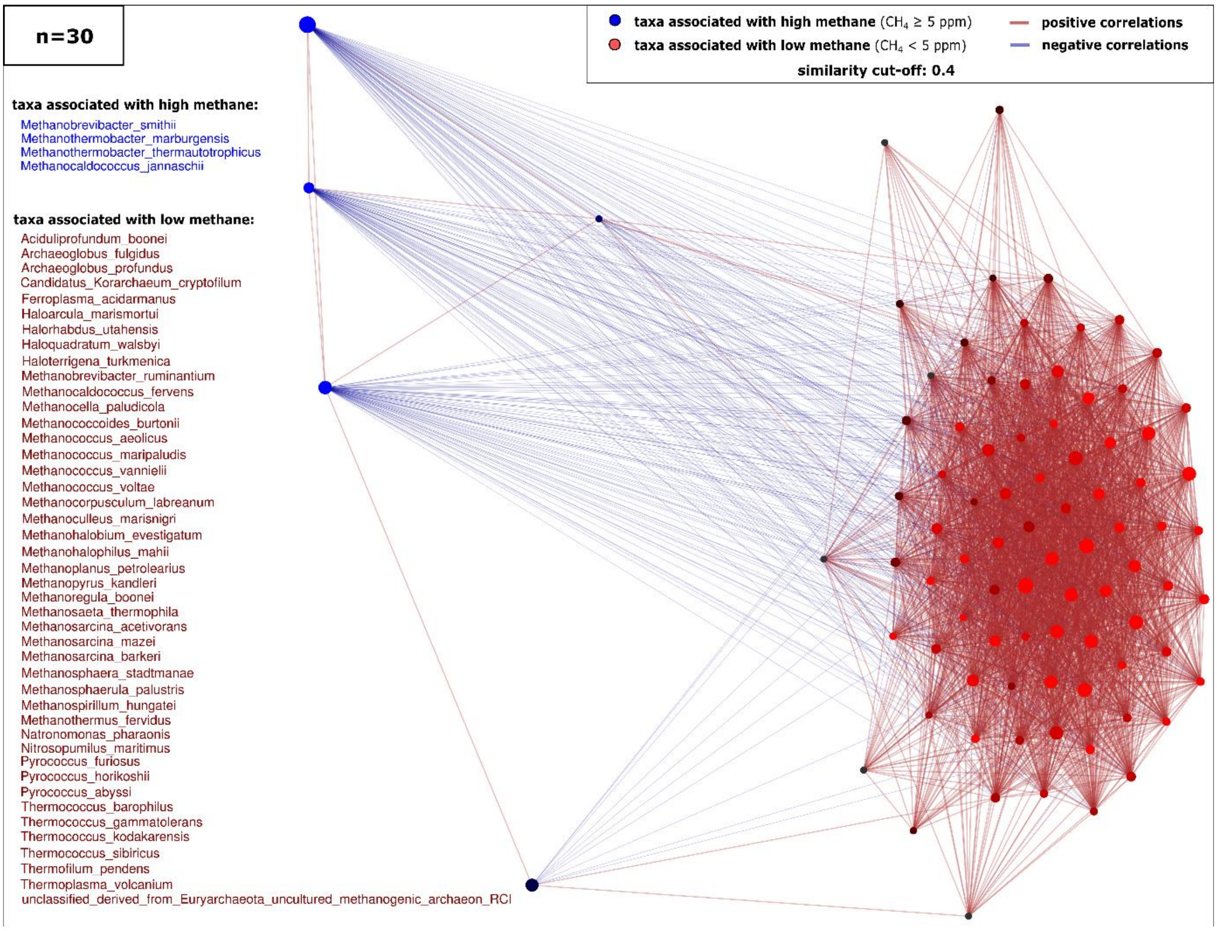

Supplementary Figure 12. Archaeal network in LE and HE (blue) based on shotgun metagenomics information (RefSeq).

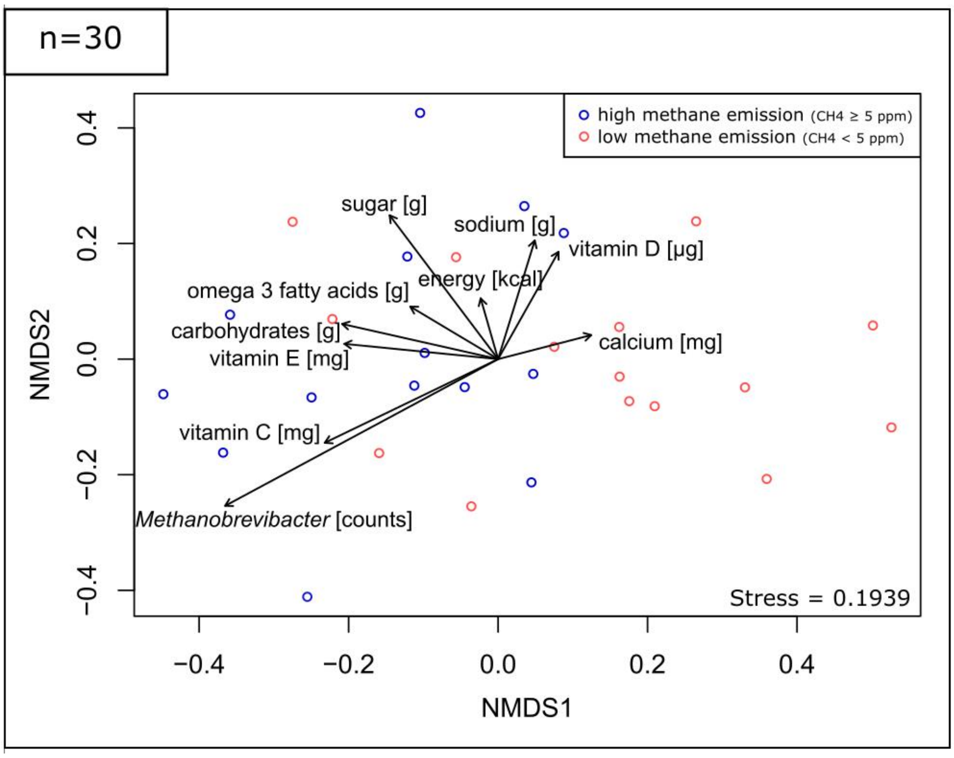

Supplementary Figure 13. Correlations with dietary intake. BIOENV analysis showing explanatory variables triggering the microbial communities of HEs (blue) and LEs (red) based on Euclidean distances that were superimposed on a Non-metric multidimensional scaling (NMDS) plot derived from Bray-Curtis dissimilarities of HE and LE samples (stress:0.1939). *Methanobrevibacter* read counts were included as a variable for better orientation.
