# Supplementary material for: Reduced B12 uptake and increased gastrointestinal formate are associated with archaeome-mediated breath methane emission in humans
Source: Microbiome. 2021 Sep 24;9:193. doi: 10.1186/s40168-021-01130-w (PMC8464155; doi:10.1186/s40168-021-01130-w)
Supplement: Supplementary file 4 — Additional file 3: Supplementary Items 6-13. Supplementary Item 6. Heatmap of amino acid flux predictions according to MICOM (universal primer: 515F-806R; n=30). Supplementary Item 7. Heatmap of C1-C4 flux predictions according to MICOM (universal primer: 515F-806R; n=30). Supplementary Item 8. Heatmap of complex compound flux predictions according to MICOM (universal primer: 515F-806R; n=30). Supplementary Item 9. Heatmap of fat flux predictions according to MICOM (universal primer: 515F-806R; n=30). Supplementary Item 10. Heatmap of nucleotide flux predictions according to MICOM (universal primer: 515F-806R; n=30). Supplementary Item 11. Heatmap of other metabolite flux predictions according to MICOM (universal primer: 515F-806R; n=30). Supplementary Item 12. Heatmap of sugar flux predictions according to MICOM (universal primer: 515F-806R; n=30). Supplementary Item 13. Heatmap of vitamine flux predictions according to MICOM (universal primer: 515F-806R; n=30). [file 40168_2021_1130_MOESM4_ESM.pdf]

Predicted exchange fluxes of amino acids from high (HE) and low (LE) methane emitters

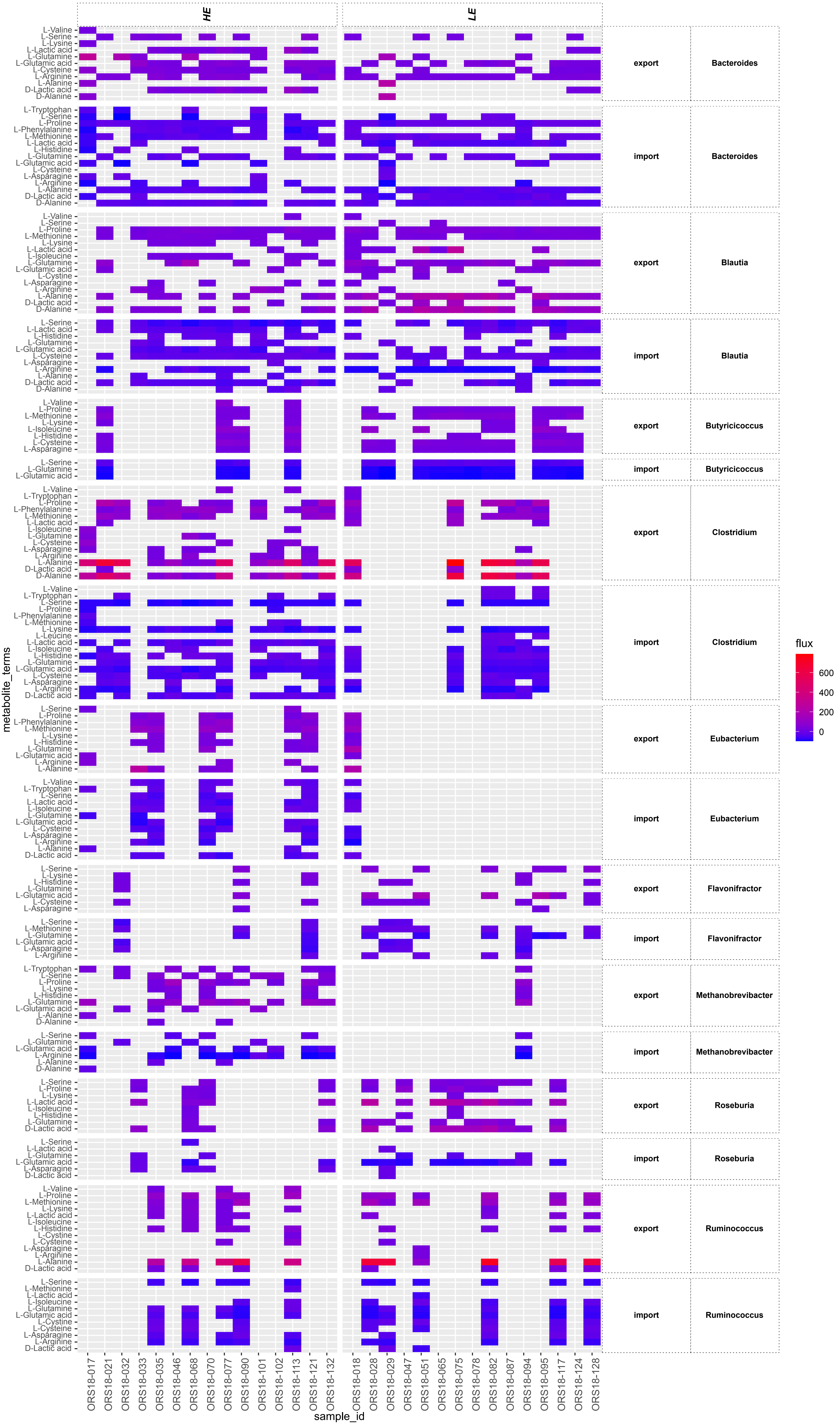

Predicted exchange fluxes of C1-C4 metabolism from high (HE) and low (LE) methane emitters

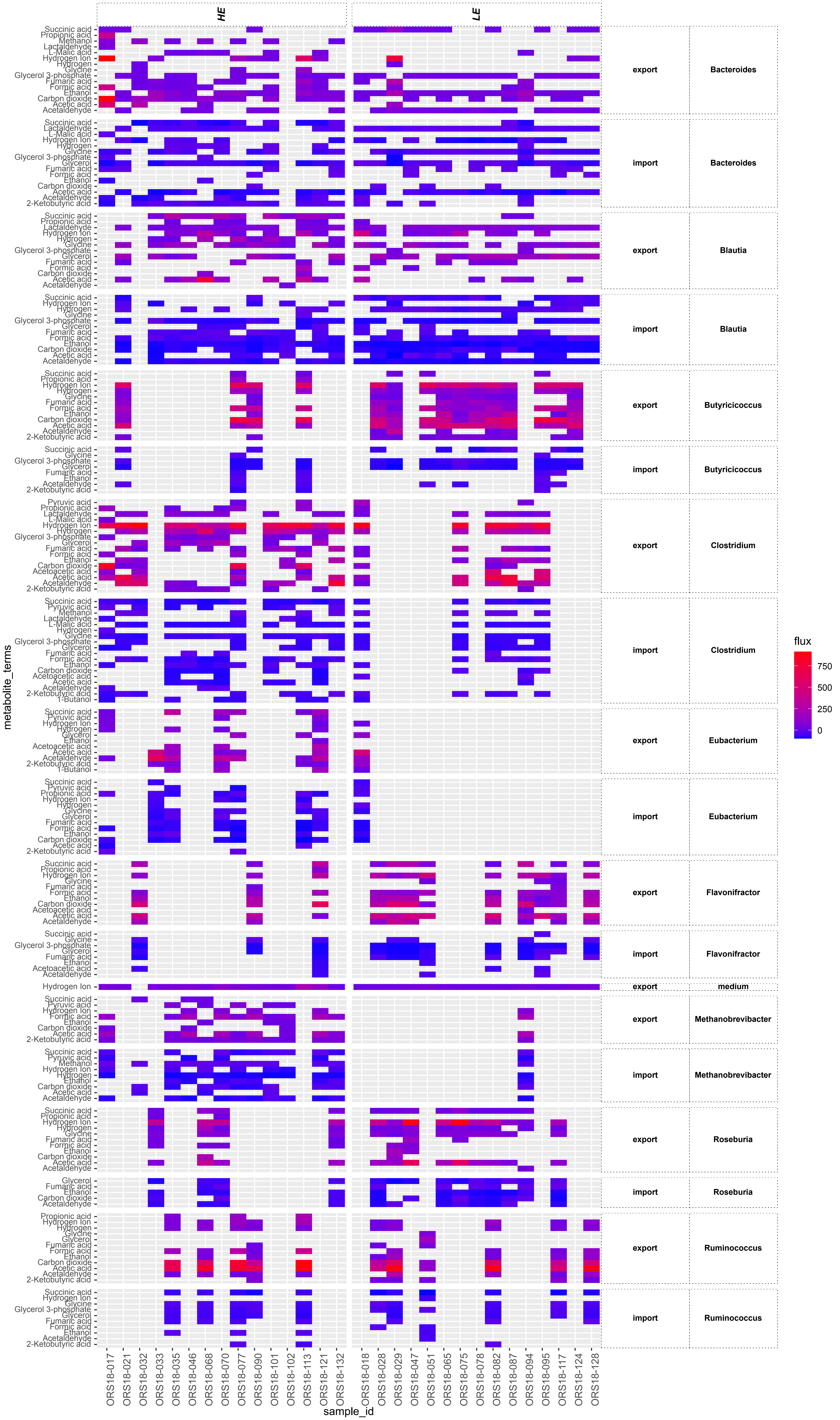

Predicted exchange fluxes of complex compounds from high (HE) and low (LE) methane emitters

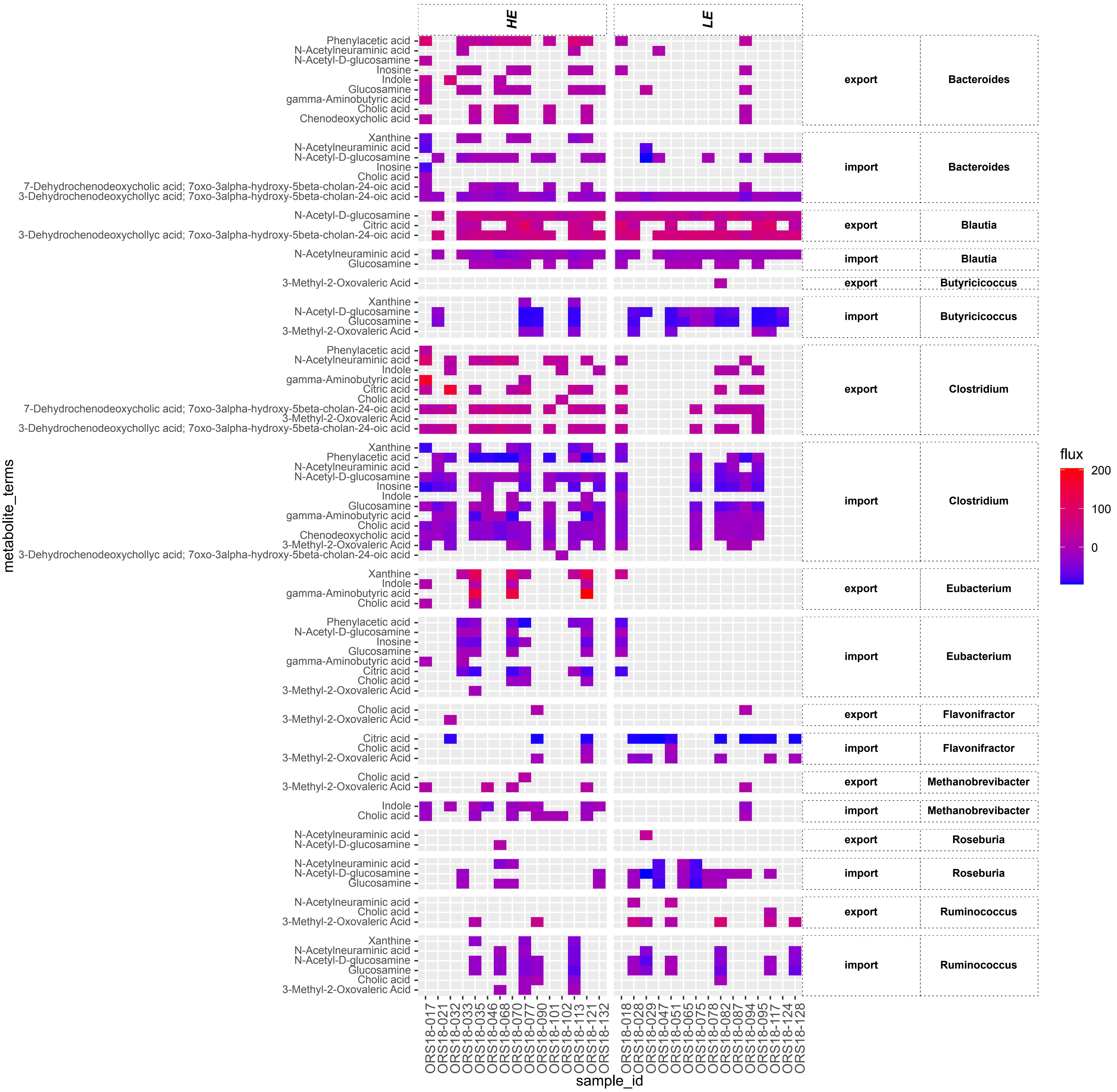

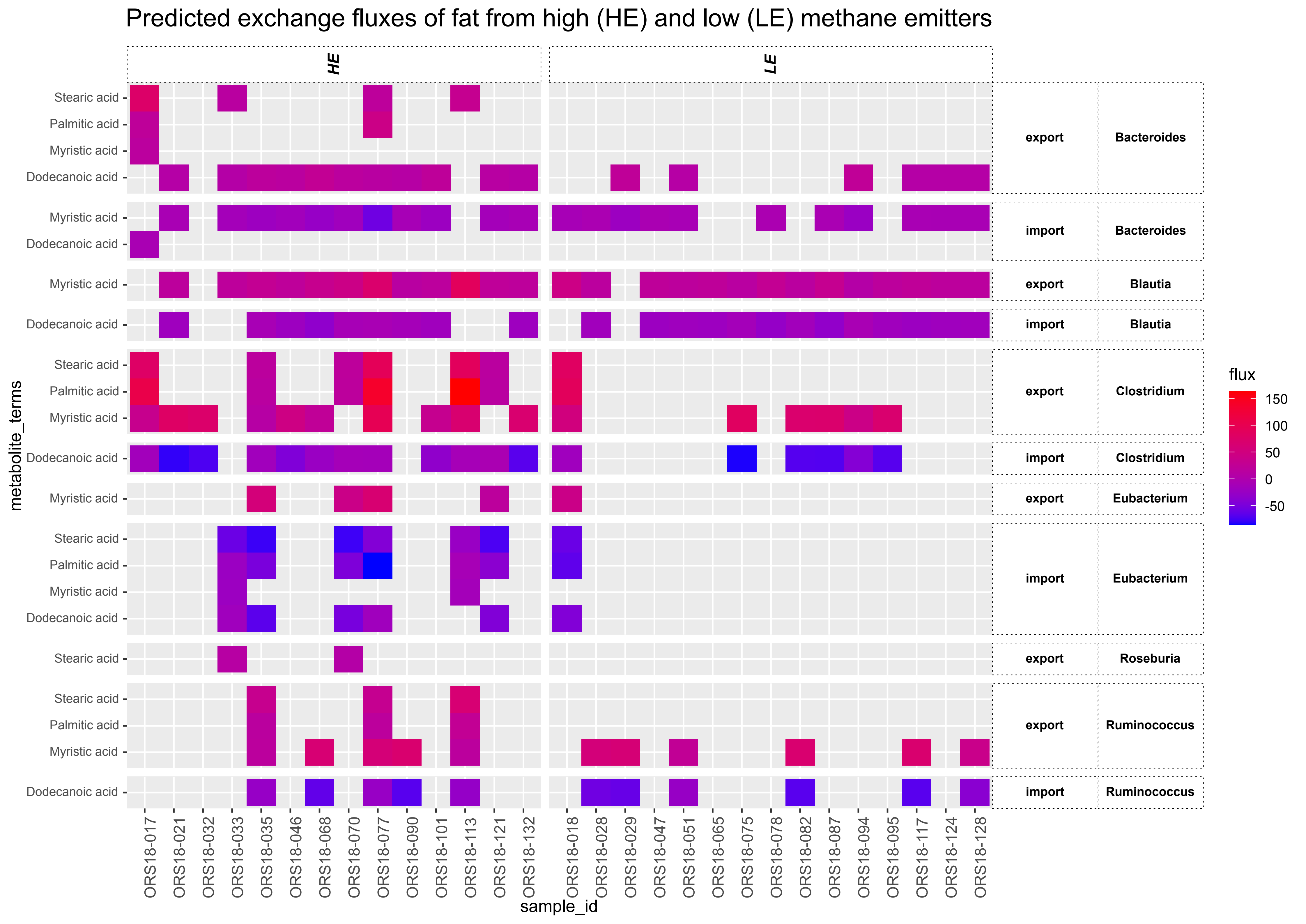

Predicted exchange fluxes of fat from high (HE) and low (LE) methane emitters
HE
LE
Stearic acid
Palmitic acid
Myristic acid
Dodecanoic acid
export
import
Bacteroides
Blautia
Clostridium
Eubacterium
Roseburia
Ruminococcus
metabolite_terms
sample_id
ORS18-017
ORS18-021
ORS18-032
ORS18-033
ORS18-035
ORS18-046
ORS18-068
ORS18-070
ORS18-077
ORS18-090
ORS18-101
ORS18-113
ORS18-121
ORS18-132
ORS18-018
ORS18-028
ORS18-029
ORS18-047
ORS18-051
ORS18-065
ORS18-075
ORS18-078
ORS18-082
ORS18-087
ORS18-094
ORS18-095
ORS18-117
ORS18-124
ORS18-128
flux
150
100
50
0
-50

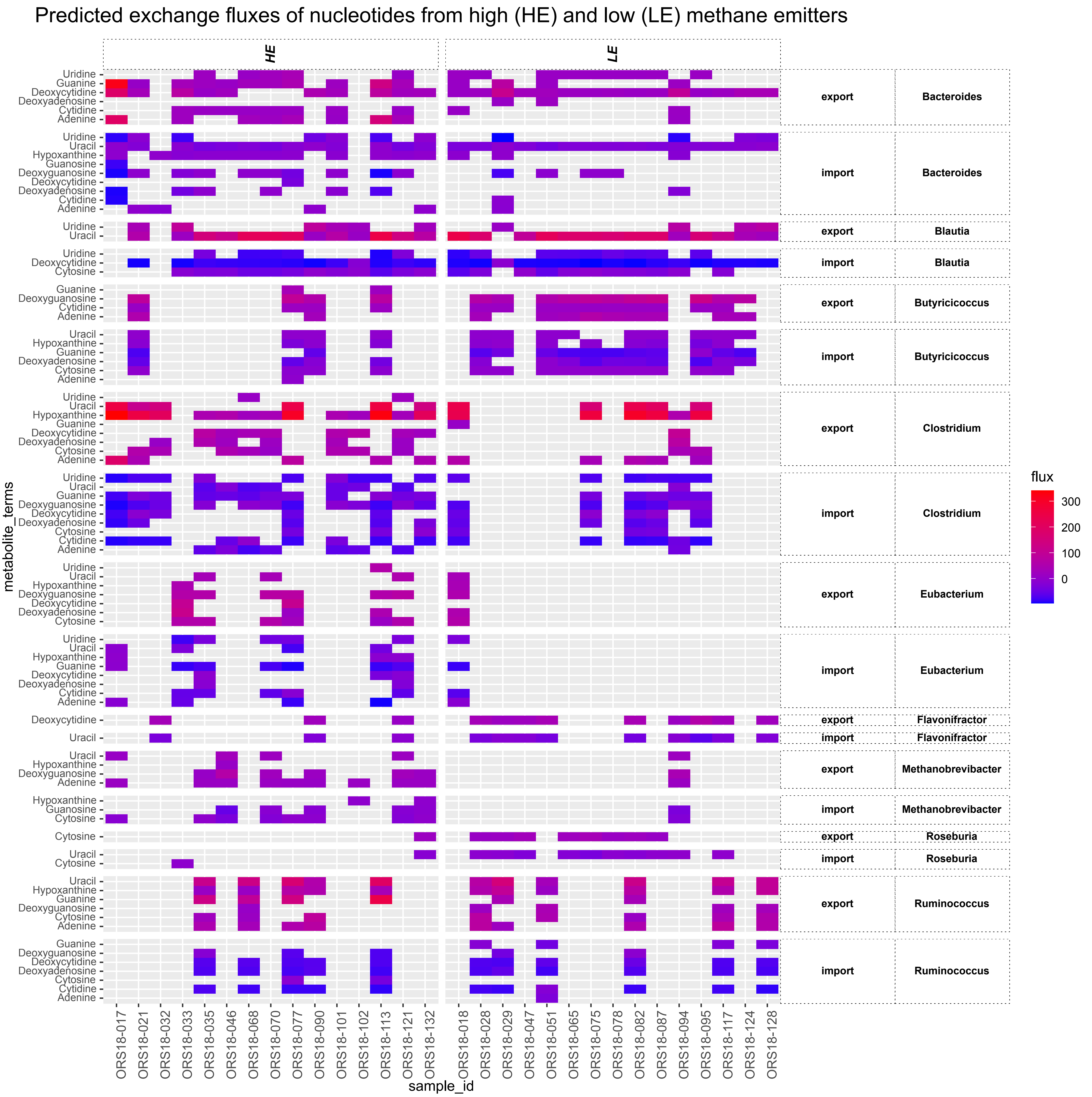
Predicted exchange fluxes of nucleotides from high (HE) and low (LE) methane emitters
HE
LE
metabolite_terms
sample_id
flux
export
import
Bacteroides
Blautia
Butyricicoccus
Clostridium
Eubacterium
Flavonifractor
Methanobrevibacter
Roseburia
Ruminococcus
Uridine
Guanine
Deoxycytidine
Deoxyadenosine
Cytidine
Adenine
Uracil
Hypoxanthine
Guanosine
Deoxyguanosine
Cytosine
ORS18-017
ORS18-021
ORS18-032
ORS18-033
ORS18-035
ORS18-046
ORS18-068
ORS18-070
ORS18-077
ORS18-090
ORS18-101
ORS18-102
ORS18-113
ORS18-121
ORS18-132
ORS18-018
ORS18-028
ORS18-029
ORS18-047
ORS18-051
ORS18-065
ORS18-075
ORS18-078
ORS18-082
ORS18-087
ORS18-094
ORS18-095
ORS18-117
ORS18-124
ORS18-128
300
200
100
0

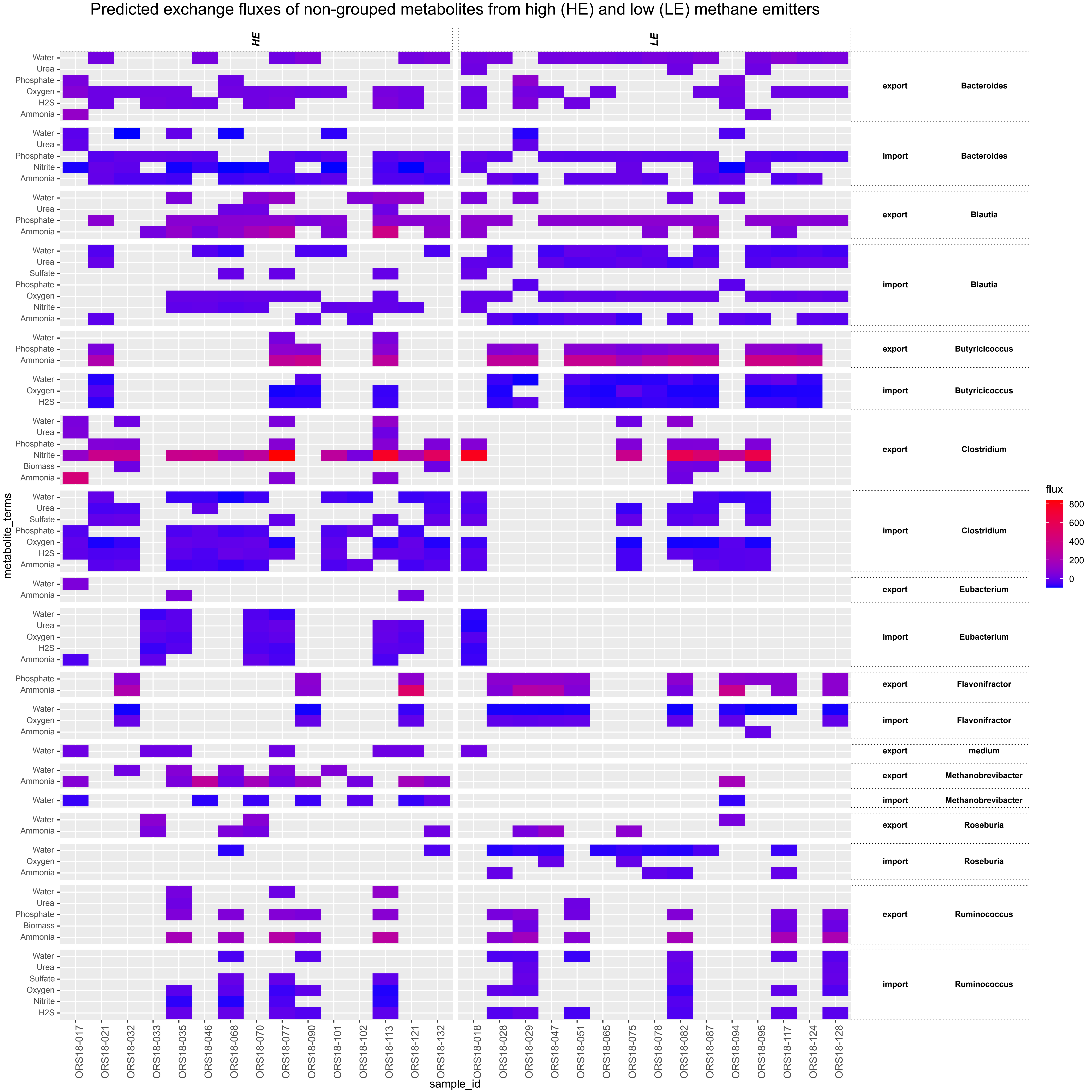

Predicted exchange fluxes of non-grouped metabolites from high (HE) and low (LE) methane emitters
HE
LE
metabolite_terms
Water
Urea
Phosphate
Oxygen
H2S
Ammonia
Nitrite
Sulfate
Biomass
export
import
Bacteroides
Blautia
Butyricicoccus
Clostridium
Eubacterium
Flavonifractor
medium
Methanobrevibacter
Roseburia
Ruminococcus
ORS18-017
ORS18-021
ORS18-032
ORS18-033
ORS18-035
ORS18-046
ORS18-068
ORS18-070
ORS18-077
ORS18-090
ORS18-101
ORS18-102
ORS18-113
ORS18-121
ORS18-132
ORS18-018
ORS18-028
ORS18-029
ORS18-047
ORS18-051
ORS18-065
ORS18-075
ORS18-078
ORS18-082
ORS18-087
ORS18-094
ORS18-095
ORS18-117
ORS18-124
ORS18-128
sample_id
flux
800
600
400
200
0

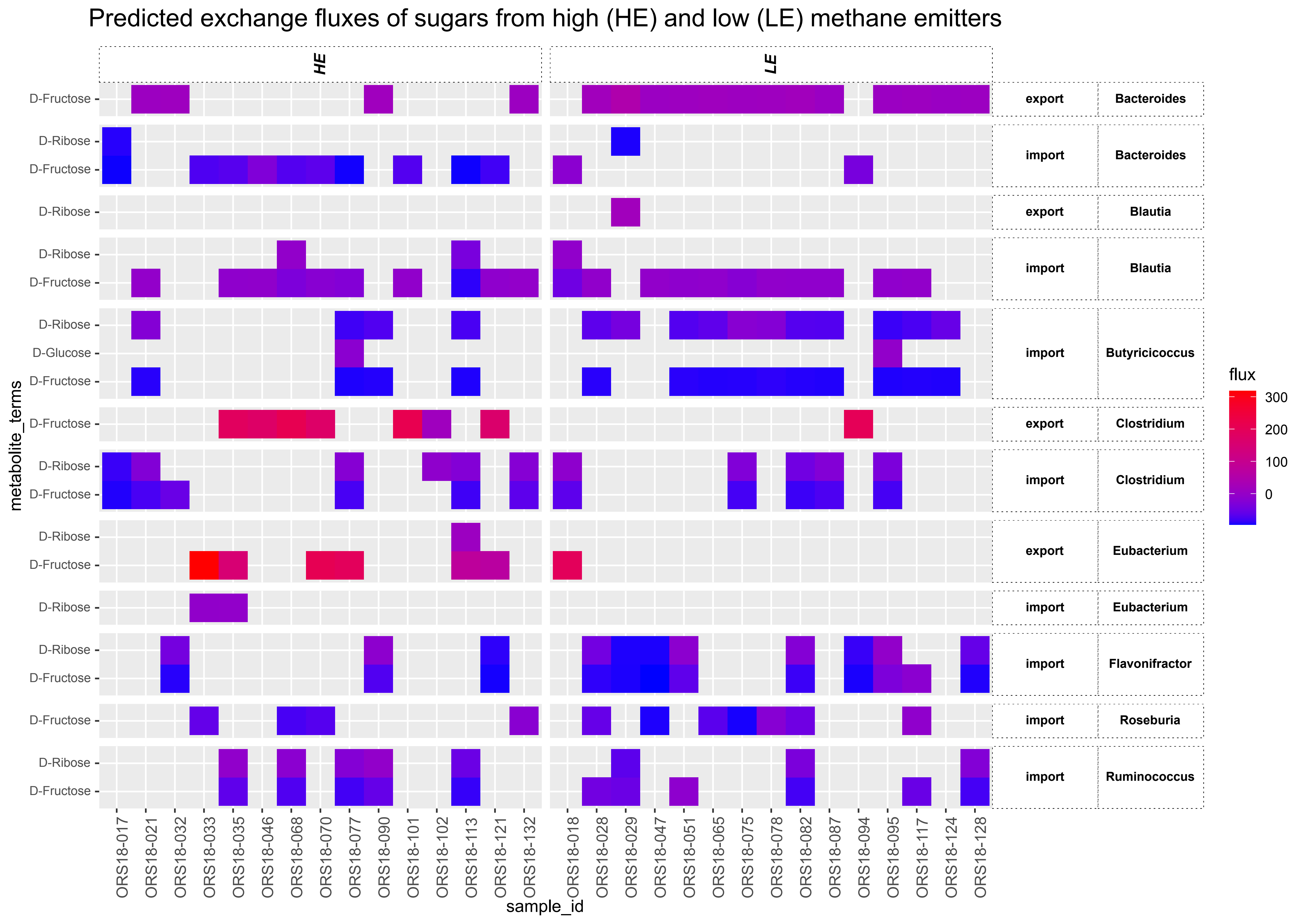

Predicted exchange fluxes of sugars from high (HE) and low (LE) methane emitters
HE
LE
metabolite_terms
sample_id
D-Fructose
D-Ribose
D-Glucose
export
import
Bacteroides
Blautia
Butyricicoccus
Clostridium
Eubacterium
Flavonifractor
Roseburia
Ruminococcus
flux
300
200
100
0
ORS18-017
ORS18-021
ORS18-032
ORS18-033
ORS18-035
ORS18-046
ORS18-068
ORS18-070
ORS18-077
ORS18-090
ORS18-101
ORS18-102
ORS18-113
ORS18-121
ORS18-132
ORS18-018
ORS18-028
ORS18-029
ORS18-047
ORS18-051
ORS18-065
ORS18-075
ORS18-078
ORS18-082
ORS18-087
ORS18-094
ORS18-095
ORS18-117
ORS18-124
ORS18-128

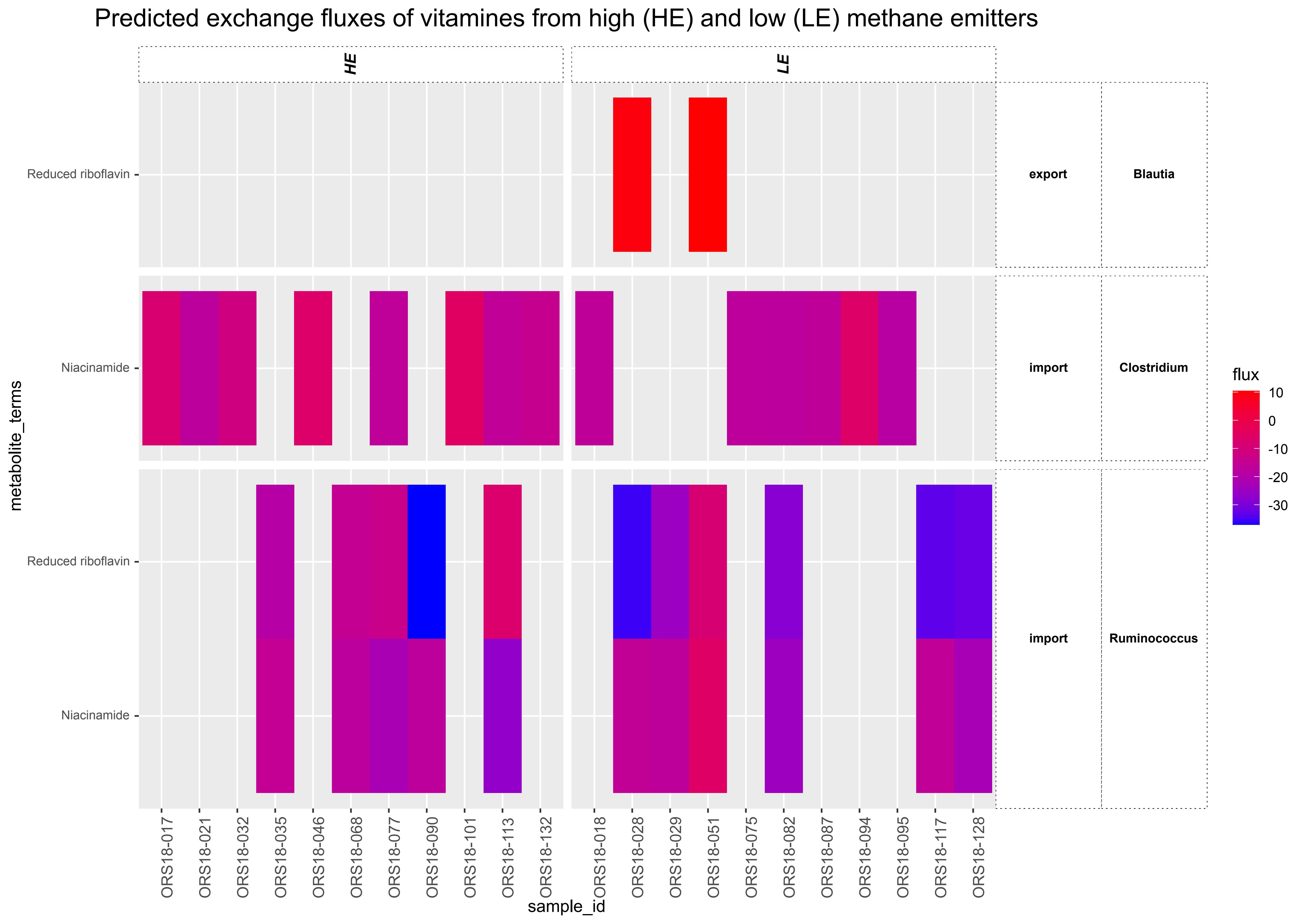

Predicted exchange fluxes of vitamines from high (HE) and low (LE) methane emitters
HE
LE
Reduced riboflavin
Niacinamide
Reduced riboflavin
Niacinamide
export
Blautia
import
Clostridium
import
Ruminococcus
metabolite_terms
flux
10
0
-10
-20
-30
ORS18-017
ORS18-021
ORS18-032
ORS18-035
ORS18-046
ORS18-068
ORS18-077
ORS18-090
ORS18-101
ORS18-113
ORS18-132
ORS18-018
ORS18-028
ORS18-029
ORS18-051
ORS18-075
ORS18-082
ORS18-087
ORS18-094
ORS18-095
ORS18-117
ORS18-128
sample_id
