# Supplementary material for: Reduced B12 uptake and increased gastrointestinal formate are associated with archaeome-mediated breath methane emission in humans
Source: Microbiome. 2021 Sep 24;9:193. doi: 10.1186/s40168-021-01130-w (PMC8464155; doi:10.1186/s40168-021-01130-w)
Supplement: Supplementary file 11 — Additional file 10. [file 40168_2021_1130_MOESM11_ESM.pdf]

Supplementary Methods

Reduced B12 uptake and increased gastrointestinal formate drive archaeome-mediated breath methane emission in humans

Short title: B12 shortage, fibre and formate drive breath methane emission

Christina Kumpitsch,[1] Florian Ph. S. Fischmeister,[2,3] Alexander Mahnert,[1] Sonja Lackner,[4] Marilena Wilding,[2] Corina Sturm,[2] Anna Springer,[5] Tobias Madl,[5,6] Sandra Holasek,[4] Christoph Högenauer,[7] Ivan Berg,[8] Veronika Schoepf,[3] Christine Moissl-Eichinger[1,6*]

Key Resources Table

REAGENT or RESOURCE	SOURCE	IDENTIFIER
**Dietary information**		
German Food Frequency Questionnaire (FFG)	[1]	Robert Koch Institute
**Methane measurement**		
GastroCH_4ECK breath bags	Bedfont Scientific Ltd, UK	#GASTROCHECK-BAG-XL
GastroCH_4ECK Gastrolyzer	Bedfont Scientific Ltd, UK	NA.
**Commercial Kit**		
PowerSoil® DNA Isolation Kit	QIAGEN, USA	#12888-100
**PMA treatment**		
PMA solution (20 mM)	VWR	#40019
PMA-Lite™ LED Photolysis Device	Biotum	NA
**Cell disruption and DNA conc. measurement**		
MagNaLyser	MagNaLyser	NA
Qubit fluorometer 2.0	Thermo Fisher Scientific, USA	NA
Qubit dsDNA HS Assay Kit	Thermo Fisher Scientific, USA	#Q32854

**PCR Reagents**		
TAKARA Ex Taq® buffer with $MgCl_2$ (10x)	Takara Bio Inc.	#RR001A
dNTP mix (200 µM)	Takara Bio Inc.	#RR001A
TAKARA Ex Taq® Polymerase (0.5 U)	Takara Bio Inc.	#RR001A
PCR grade water	Jena Bioscience, Germany	#PCR-258S
**Oligonucleotides**		
Universal forward primer: Illu-515FB TCGTCGGCAGCGTCAGATGTGTATAAGAGACAGGTG **YCAGCMGCCGCGGTAA**	[2]	Eurofins Genomics AT GmbH, Austria
Universal reverse primer: Illu-806RB GTCTCGTGGGCTCGGAGATGTGTATAAGAGACAGGG **ACTACNVGGGTWTCTAAT**	[2]	Eurofins Genomics AT GmbH, Austria
Archaeal forward primer (nested PCR1): 344F ACGGGGYGCAGCAGGCGCGA	[2]	Eurofins Genomics AT GmbH, Austria
Archaeal reverse primer (nested PCR1): 1041R ACGGGGYGCAGCAGGCGCGA	[2]	Eurofins Genomics AT GmbH, Austria
Archaeal forward primer (nested PCR2): 519F TCGTCGGCAGCGTCAGATGTGTATAAGAGACAGCA **GCMGCCGCGGTAA**	[2]	Eurofins Genomics AT GmbH, Austria
Archaeal reverse primer (nested PCR2): 806R GTCTCGTGGGCTCGGAGATGTGTATAAGAGACAGGG **ACTACVSGGGTATCTAAT**	[2]	Eurofins Genomics AT GmbH, Austria
Bacterial primer (qPCR): 331F TCCTACGGGAGGCAGCAGT	[3]	Eurofins Genomics AT GmbH, Austria
Bacterial primer (qPCR): 797R GGACTACCAGGGTATCTAATCCTGTT	[3]	Eurofins Genomics AT GmbH, Austria
mcrA primer (qPCR): M1F GCAATGCAAATTGGTATGTC	Modified from this paper [4]	Eurofins Genomics AT GmbH, Austria
mcrA primer (qPCR): M1R TCATTGCGTAGTTAGGRTAGT	Modified from this paper [4]	Eurofins Genomics AT GmbH, Austria

**Illumina sequencing**		
Illumina MiSeq sequencing platform	Illumina, Eindhoven, the Netherlands	Core Facility for Molecular Biology (Center for Medical Research, Graz)<br>[5]
Nextera XT Library construction kit	Illumina, Eindhoven, the Netherlands	Macrogen (Seoul, South Korea)
Illumina HiSeq technique	Illumina, Eindhoven, the Netherlands	Macrogen (Seoul, South Korea)
**Software and Algorithms**		
QIIME2 version 2020.6	[6]	https://qiime2.org
SILVA version 128-132	[7]	
R version 3.5.1<br>R Studio version 1.2.1335	[8]	https://www.r-project.org
decontam version 1.1.0	[9]	https://github.com/benjjneb/decontam
vegan version 2.5-5	[10]	
Calypso	[11]	http://cgenome.net:8080/calypso-8.84/faces/uploadFiles.xhtml
Galaxy set-up	[12]	provided by the Core Facility Computational Biology at the Medical University of Graz
LEfSe	[13]	http://huttenhower.sph.harvard.edu/galaxy/
Krona chart	[14]	Krona chart excel template
nut.s - nutritional software version 1.32.79	[15]	www.nutritional-software.at
MG-Rast platform (server running version 4.0.3.)	[16]	https://www.mg-rast.org/
IBM SPSS Amos version 26	IBM	http://www.ibm.com/analytics/us/en/technology/spss/
fastqc v0.11.8	[17]	http://www.bioinformatics.babraham.ac.uk/projects/fastqc/
trimmomatic v0.38	[18]	http://www.usadellab.org/c

		ms/?page=trimmomatic
bowtie2 v2.3.5	[19]	http://bowtie-bio.sourceforge.net/bowtie2/index.shtml
samtools v1.9	[20]	http://www.htslib.org/
bedtools v2.29.0	[21]	https://bedtools.readthedocs.io/en/latest/
diamond v0.9.25	[22]	http://www.diamondsearch.org/index.php
MEGAN v6.20.13	[23]	https://software-ab.informatik.uni-tuebingen.de/download/megan6/welcome.html
Megahit v1.1.3	[24]	https://github.com/voutcn/megahit
MaxBin v2.2.4	[25]	https://sourceforge.net/projects/maxbin2/
dRep v2.0.5	[26]	https://github.com/MrOlm/drep
GTDBtk v1.2.0	[27]	https://github.com/Ecogenomics/GTDBTk
MaGe	[28]	https://mage.genoscope.cns.fr/microscope/home/index.php
iRep v1.1.9	[29]	https://github.com/christophertbrown/iRep
q2-sample-classifier-plugin	[30]	https://docs.qiime2.org/2020.11/tutorials/sample-classifier/
q2-micom plugin v.0.8.0	[31]	https://github.com/micom-dev/q2-micom
AGORA genus model database v1.03	[32]	https://www.vmh.life/#downloadview
**Deposited Data**		
Raw data (amplicon, metagenome)	European Nucleotide Archive (ENA)	PRJEB41867
Supplementary Dataset (Sequencing data after decontam and removal of	Mendeley data [33]	http://dx.doi.org/10.17632/hjj3tx7n84.1

features with zero and one reads) Supplementary Figures, Tables and Items		

PCR conditions (amplicon):

	universal	Archaea	
		(nested PCR 1)	(nested PCR2)
**Initial denaturation**	94°C, 3´	95°C, 5´	95°C, 5´
**Denaturation**	94°C, 45´´	94°C, 30´´	95°C, 40´´
**Annealing**	50°C, 60´´	56°C, 45´´	63°C, 2´
**Elongation**	72°C, 1´30´´	72°C, 1´	72°C, 1´
**Final elongation**	72°C, 10´	72°C, 10´	72°C, 10´
**No. of cycles**	**35**	**25**	**30**

PCR reagents and conditions (qPCR):

component	final conc.
**SsoAdvance Universal SYBR**	1X
**Forward primer A806F**	300 nM
**Reverse primer A958R**	300 nM
**Template - archaea**	1µl
**Water**	fill up to 10µl
**Final Volume: 10µl**	

	bacteria	methanogens
**Initial denaturation**	95°C, 15´	94°C, 3´
**Denaturation**	94°C, 15´´	94°C, 45´´
**Annealing**	54°C, 30´´	56°C, 45´´
**Elongation**	72°C, 40´´	72°C, 30´
**No. of cycles**	**40**	**40**
**Melting curve**	**60-95°C**	**60-95°C**

References

1. Haftenberger M, Heuer T, Heidemann C, Kube F, Krems C, Mensink GBM. Relative validation of a food frequency questionnaire for national health and nutrition monitoring. Nutr J. Nutr J; 2010;9.

2. Pausan MR, Csorba C, Singer G, Till H, Schoepf V, Santigli E, et al. Exploring the Archaeome: Detection of Archaeal Signatures in the Human Body. Front Microbiol. 2019;10:2796.

3. Probst AJ, Auerbach AK, Moissl-Eichinger C. Archaea on Human Skin. PLoS One. 2013;8.

4. Hales BA, Edwards C, Ritchie DA, Hall G, Pickup RW, Saunders JR. Isolation and identification of methanogen-specific DNA from blanket bog peat by PCR amplification and sequence analysis. Appl Environ Microbiol. 1996;62:668–75.

5. Klymiuk I, Bambach I, Patra V, Trajanoski S, Wolf P. 16S based microbiome analysis from healthy subjects' skin swabs stored for different storage periods reveal phylum to genus level changes. Front Microbiol. Frontiers Media SA; 2016;7.

6. Bolyen E, Rideout JR, Dillon MR, Bokulich NA, Abnet CC, Al-Ghalith GA, et al. Reproducible, interactive, scalable and extensible microbiome data science using QIIME 2. Nat. Biotechnol. Nature Publishing Group; 2019. p. 852–7.

7. Quast C, Pruesse E, Yilmaz P, Gerken J, Schweer T, Yarza P, et al. The SILVA ribosomal RNA gene database project: improved data processing and web-based tools. Nucleic Acids Res. 2013;41:D590–6.

8. Team RDC. R: A Language and Environment for Statistical Computing. 2019.

9. Davis NM, Proctor DM, Holmes SP, Relman DA, B.J C. Simple statistical identification and removal of contaminant sequences in marker-gene and metagenomics data. Microbiome. 2018;6:226.

10. Oksanen J, Kindt R, Legendre P, O'Hara B, Henry M, Stevens H. The vegan package -Community ecology package. 2007. p. 631–7.

11. Zakrzewski M, Proietti C, Ellis JJ, Hasan S, Brion M-J, Berger B KL. Calypso: a user-friendly web-server for mining and visualizing microbiome-environment interactions. Bioinformatics. 2017;33:782–3.

12. Jalili V, Afgan E, Gu Q, Clements D, Blankenberg D, Goecks J, et al. The Galaxy platform for accessible, reproducible and collaborative biomedical analyses: 2020 update. Nucleic Acids Res. NLM (Medline); 2020;48:W395–402.

13. Segata N, Izard J, Waldron L, Gevers D, Miropolsky L, Garrett WS, et al. Metagenomic biomarker discovery and explanation. Genome Biol [Internet]. BioMed Central; 2011;12:1.

14. Ondov BD, Bergman NH, Phillippy AM. Interactive metagenomic visualization in a Web browser. BMC Bioinformatics. 2011;12:1.

15. Denkwerkzeuge D. Software:nut.s science v1.32.79. Vienna; 2020.

16. Meyer F, Paarmann D, D'Souza M, Olson R, Glass EM, Kubal M, et al. The metagenomics RAST server - A public resource for the automatic phylogenetic and functional analysis of metagenomes. BMC Bioinformatics. BMC Bioinformatics; 2008;9.
17. Andrews S. FastQC: a quality control tool for high throughput sequence data. 2010.
18. Bolger AM, Lohse M, Usadel B. Trimmomatic: a flexible trimmer for Illumina sequence data. Bioinformatics. Oxford University Press; 2014;30:2114–20.
19. Langmead B, Salzberg SL. Fast gapped-read alignment with Bowtie 2. Nat Methods. Nature Publishing Group; 2012;9:357.
20. Li H, Handsaker B, Wysoker A, Fennell T, Ruan J, Homer N, et al. The sequence alignment/map format and SAMtools. Bioinformatics. Oxford University Press; 2009;25:2078–9.
21. Quinlan AR, Hall IM. BEDTools: A flexible suite of utilities for comparing genomic features. Bioinformatics. Bioinformatics; 2010;26:841–2.
22. Buchfink B, Xie C, Huson DH. Fast and sensitive protein alignment using DIAMOND. Nat Methods. Nature Publishing Group; 2015;12:59.
23. Huson DH, Auch AF, Qi J, Schuster SC. MEGAN analysis of metagenomic data. Genome Res. 2007;17:377–86.
24. Li D, Liu CM, Luo R, Sadakane K, Lam TW. MEGAHIT: An ultra-fast single-node solution for large and complex metagenomics assembly via succinct de Bruijn graph. Bioinformatics. Oxford University Press; 2015;31:1674–6.
25. Wu YW, Tang YH, Tringe SG, Simmons BA, Singer SW. MaxBin: An automated binning method to recover individual genomes from metagenomes using an expectation-maximization algorithm. Microbiome. BioMed Central Ltd.; 2014;2.
26. Olm MR, Brown CT, Brooks B, Banfield JF. DRep: A tool for fast and accurate genomic comparisons that enables improved genome recovery from metagenomes through de-replication. ISME J. Nature Publishing Group; 2017;11:2864–8.
27. Chaumeil PA, Mussig AJ, Hugenholtz P, Parks DH. GTDB-Tk: A toolkit to classify genomes with the genome taxonomy database. Bioinformatics. Oxford University Press; 2020;36:1925–7.
28. Vallenet D, Labarre L, Rouy Z, Barbe V, Bocs S, Cruveiller S, et al. MaGe: A microbial genome annotation system supported by synteny results. Nucleic Acids Res. 2006;34:53–65.
29. Brown CT, Olm MR, Thomas BC, Banfield JF. Measurement of bacterial replication rates in microbial communities. Nat Biotechnol. Nature Publishing Group; 2016;34:1256.
30. Bokulich N, Dillon M, Bolyen E, Kaehler B, Huttley G, Caporaso J. q2-sample-classifier: machine-learning tools for microbiome classification and regression. J Open Source Softw. The Open Journal; 2018;3:934.
31. Diener C, Gibbons SM, Resendis-Antonio O. MICOM: Metagenome-Scale Modeling To Infer

Metabolic Interactions in the Gut Microbiota. mSystems. American Society for Microbiology; 2020;5.
32. Magnúsdóttir S, Heinken A, Kutt L, Ravcheev DA, Bauer E, Noronha A, et al. Generation of genome-scale metabolic reconstructions for 773 members of the human gut microbiota. Nat Microbiol. 2017;35:81–89.
33. Kumpitsch C, Fischmeister F, Mahnert A, Lackner S, Wilding M, Sturm C, et al. “Methane emission of humans is explained by dietary habits, host genetics, local formate availability and a uniform archaeome”, Mendeley Data. 2020.
